# METTL14‐Mediated M^6^A Modification of LINC01094 Induces Glucose Metabolic Reprogramming in Breast Cancer by Recruiting the PKM2/JMJD5 Complex

**DOI:** 10.1002/advs.202410386

**Published:** 2025-06-05

**Authors:** Mengqi Wang, Zhaoxin Gao, Ruinan Zhao, Pan Zhou, Jingxi Chen, Hui Zhang, Yawen Wang, Wenjie Zhu, Peng Gao

**Affiliations:** ^1^ Department of Pathology Qilu Hospital and School of Basic Medical Sciences Shandong University Jinan 250000 China; ^2^ Department of Obstetrics Qilu Hospital Shandong University Jinan 250000 China

**Keywords:** LINC01094, PKM2, breast cancer, glucose metabolic reprogramming, m^6^A modification

## Abstract

Tumor cells reprogram their energy metabolism patterns to meet the needs of rapid growth and metastasis. The underlying mechanisms of long noncoding RNAs (lncRNAs) in glucose metabolism remodeling in breast cancer (BC) are still not well understood. Herein, the expression of a tumorigenic lncRNA, LINC01094 are demonstrated that, is significantly increased in BC tissues and is associated with poorer patient survival. METTL14‐mediated m^6^A modification stabilized LINC01094 by recruiting the reader protein IGF2BP2, which contributed to the upregulation of LINC01094 expression in BC. Gain‐ and loss‐of‐function assays validated that LINC01094 triggered a switch in glucose metabolism from mitochondrial respiration to glycolysis, promoting BC progression both in vitro and in vivo. LINC01094 promoted the dimeric assembly and nuclear translocation of PKM2 by acting as a “molecular scaffold” for the PKM2/JMJD5 complex. This, in turn, facilitated energy metabolic reprogramming and cell proliferation induced by HIF1‐α/β‐catenin. Furthermore, the therapeutic potential of LINC01094 is evaluated through the administration of the PKM2 activator TEPP‐46 in mouse xenografts. These findings highlight the critical roles of LINC01094 in cellular glucose metabolism and tumorigenesis in BC, suggesting that it is a potential therapeutic target.

## Graphical abstract

METTL14/IGF2BP2‐mediated m^6^A modification drives LINC01094 upregulation in BC. Then, LINC01094 interacts with PKM2 monomers to promote their dimerization, while serving as a flexible scaffold to facilitate the assembly of the PKM2/JMJD5 complex, synergistically stabilizing PKM2 dimers and enhancing their nuclear translocation. This dual mechanism potentiates PKM2‐induced transcriptional activity of HIF‐1α/β‐catenin, ultimately triggering a cascade in aerobic glycolytic and cancer progression.

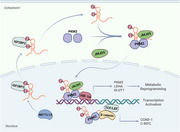

## 1 Introduction

Breast cancer (BC) is the most common malignancy worldwide.^[^ ^1^ ^]^ The morbidity and mortality of BC increase annually, making it a major threat to health.^[^ ^2^ ^]^ Patients with early non‐metastatic BC can be cured, but advanced BC with distant metastases is considered incurable.^[^ ^3^ ^]^ BC is highly heterogeneous, and the treatment strategies for BC vary according to molecular features.^[^ ^4^ ^]^ Thus, identifying relevant biomarkers and developing therapeutic targets for intervention in advanced BC are important approaches.

Most types of cancer (including BC) rely on glycolysis rather than mitochondrial oxidative phosphorylation (OXPHOS) to generate energy, even under sufficient oxygen conditions. This phenomenon is called the “Warburg effect” or “aerobic glycolysis”.^[^ ^5^ ^]^ Cancer cells alter their patterns of glucose metabolism to produce sufficient biosynthetic substrates and intermediates to facilitate their growth and progression, a process also known as “energy metabolic reprogramming”.^[^ ^6^ ^]^ Recently, energy metabolic reprogramming has been widely recognized as a central hallmark of cancer.^[^ ^7^ ^]^ Some scholars have proposed that cancer is a metabolic disease.^[^ ^8^ ^]^ Growing evidence suggests that targeting tumor metabolism may be a promising anti‐cancer therapy.^[^ ^9^ ^]^ Nonetheless, the mechanism of energy metabolic reprogramming is incompletely understood.

In BC, the dysregulation of glucose metabolism is increasingly recognized as a critical driver of disease progression, influencing not only tumor growth but also metastasis, therapeutic resistance, and immune evasion.^[^ ^10^ ^]^ Studies have shown that glycolytic flux and increased glucose uptake are associated with more aggressive subtypes of BC (e.g., triple‐negative breast cancer (TNBC)) and correlate with worse outcomes for patients.^[^ ^11^ ^]^ Targeting key enzymes in the glycolytic pathway has shown promise in preclinical models,^[^ ^12^ ^]^ but the complex regulatory networks governing these metabolic shifts are not yet fully understood.

A long non‐coding RNA (lncRNA) is >200 nucleotides in length and has limited or no potential for protein‐coding. lncRNAs play pivotal roles in regulating gene expression at the transcriptional, post‐transcriptional, and epigenetic levels by functionally acting as signal, decoy, guide, or scaffold archetypes in both *cis* and *trans* configurations.^[^ ^13^, ^14^, ^15^, ^16^ ^]^ Accumulating evidence indicates that lncRNAs are abnormally expressed in diverse types of cancer (including BC), where they act as pivotal promoters or suppressors of tumors.^[^ ^17^ ^]^ For example, the lncRNA ELFN1‐AS1 interacts with EZH2/DNMT3a complexes to promote tumorigenesis and oxaliplatin resistance in colorectal cancer by downregulating the expression of MEIS1.^[^ ^18^ ^]^ Richart et al. reported that Xist deletion inhibited stem‐cell differentiation in BC and increased tumorigenesis through hyperactivation of mediators.^[^ ^19^ ^]^ The role of lncRNAs in cancer is increasingly recognized, but their specific contribution to the metabolic reprogramming of BC remains an emerging and intensely studied area. Several lncRNAs have been implicated in regulating various aspects of cancer metabolism, including the metabolism of glucose, lipids, and glutamine. For instance, the lncRNA Neat1 has been shown to promote glycolysis in BC cells by binding to and forming a scaffold for the assembly of PGK1/PGAM1/ENO1 complexes.^[^ ^20^ ^]^ However, the vast majority of lncRNAs remain uncharacterized in the context of BC metabolic reprogramming, and the specific mechanisms through which they exert their effects are still poorly defined.

Herein, we identified and functionally characterized a novel energy metabolism‐related long intergenic non‐protein‐coding RNA 1094 (lncRNA‐LINC01094) in BC that promotes glucose metabolic flux into aerobic glycolysis. We demonstrated that LINC01094 expression was upregulated in BC by METTL14/IGF2BP2‐mediated N^6^‐methyladenosine (m^6^A) modification. LINC01094 binds to PKM2 monomers, contributing to the assembly of PKM2 dimers, as well as promoting the formation of the PKM2/JMJD5 complex by serving as a flexible scaffold. The combination of LINC01094 and PKM2 stabilizes the PKM2 dimer and promotes the entry of PKM2 into the nucleus. This phenomenon facilitates PKM2‐enhanced HIF‐1α/β‐catenin transcriptional activity and triggers a cascade reaction in aerobic glycolysis and cancer progression.

We focused on LINC01094, an lncRNA that had not been previously investigated in the context of BC metabolism. Given the general lack of understanding regarding lncRNA‐mediated metabolic regulation in BC, this observation provides a compelling rationale for a detailed investigation of the function and mechanism of action of LINC01094. The novelty of our research lies in identifying LINC01094 as a key regulator of glucose metabolism in BC, elucidating its m^6^A‐dependent upregulation, and dissecting its interaction with PKM2 and JMJD5 to promote a pro‐glycolytic and pro‐tumorigenic phenotype. The importance of this work lies in its potential to identify a novel therapeutic target for BC, particularly in aggressive subtypes that rely heavily on glycolysis for survival and proliferation.

## 2 Results

### 2.1 LINC01094 Expression is Significantly Upregulated in BC

**Figure 1:**
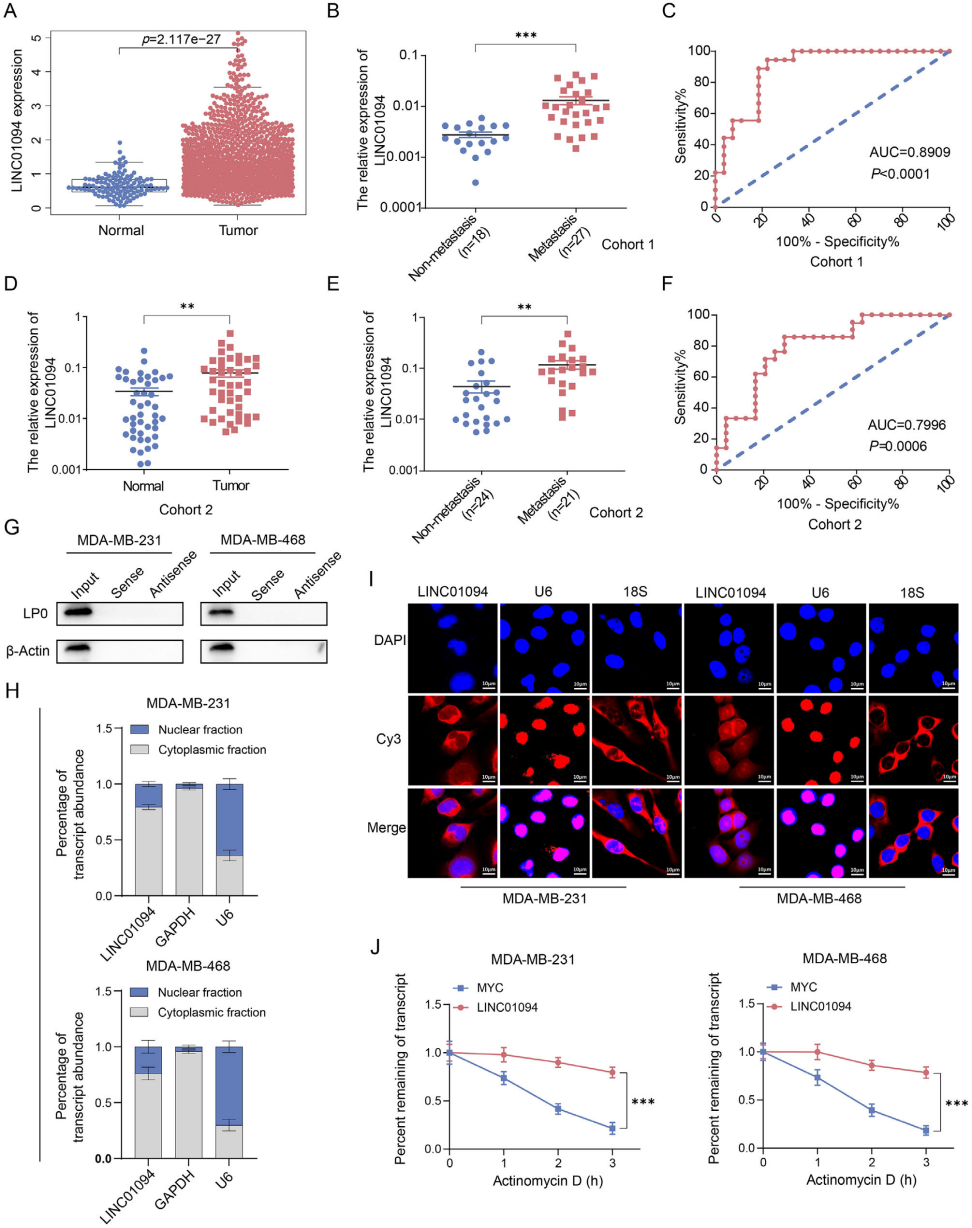
LINC01094 exhibits a significant up‐regulation in BC. A) The expression pattern of LINC01094 in BC patients comprising both tumor and normal tissues sourced from TCGA. B) qRT‐PCR analysis was conducted to investigate the correlation between LINC01094 expression and lymph node metastasis (LNM) in BC tissues from cohort 1. The expression levels of LINC01094 were normalized to GAPDH. C) The ROC curve was employed to evaluate the discriminatory potential of LINC01094 in cohort 1. D) qRT‐PCR analysis revealed a significant upregulation of LINC01094 expression in BC tissues compared to adjacent normal tissues within cohort 2. E) In cohort 2, LINC01094 expression was significantly elevated in BC tissues with LNM compared to those without LNM. F) ROC curve analysis revealed that LINC01094 served as an effective biomarker for discriminating between patients with and without LNM in cohort 2. G) Western blot analysis of pull‐down assay purifications demonstrated the interaction between LINC01094 and the ribosomal protein LP0, with β‐actin utilized as the loading control. H) Subcellular fractionation, followed by qRT‐PCR, revealed the localization of LINC01094 in BC cells. I) RNA FISH assay was conducted to identify the cellular localization of LINC01094. Scale bars: 10 µm. J) The RNA Polymerase II Inhibitor Actinomycin D (ActD) Chase assay was employed to assess the stability of LINC01094 in BC cells. Data are presented as mean ± SD; statistical significance is indicated (**p < 0.01; ***p < 0.001) by Student's t‐test or ANOVA.

We aimed to identify the lncRNAs involved in BC pathogenesis. We screened the lncRNAs that were significantly correlated with the survival of patients suffering from BC in the Cancer Genome Atlas (TCGA) dataset, among which LINC01094 was the most positively associated with worse prognosis (Figure S1A, Supporting information). Analyses of the TCGA cohort showed that LINC01094 expression was markedly upregulated in BC tissues compared to normal BC samples (**Figure** **1**A; Figure S1B, Supporting information). Next, qRT‐PCR was performed on 45 cases (cohort 1) of clinical BC tissues. LINC01094 showed significantly higher expression in the sub‐cohort with lymph‐node metastasis (LNM; *n* = 27) compared to those without LNM (*n* = 18) (Figure 1B). Analyses of receiver operating characteristic (ROC) curves revealed that LINC01094 could distinguish patients with LNM from those without LNM, with an area under the ROC curve (AUC) of 0.8909 (Figure 1C). The detailed clinicopathologic characteristics of LINC01094 are summarized in **Table** **1**.

**Table 1:** LINC01094 expression and clinicopathological features in Cohorts 1 and 2.

Clinicopathologic Features	Cohort 1[n = 45]					Cohort 2[n = 45]				
	Low LINC01094		High LINC01094		*p* value	Low LINC01094		High LINC01094		*p* value
	Case No.	[%]	Case No.	[%]		Case No.	[%]	Case No.	[%]	
**Age**										
≤35	1	4.3	0	0	0.488	0	0	1	4.5	0.382
35∼60	17	73.9	15	68.2		20	87	16	72.7	
>60	5	21.7	7	31.8		3	13	5	22.7	
**Tumor size**										
≤2	4	17.4	6	27.3	0.722	11	47.8	6	27.3	0.106
2∼5	18	78.3	15	68.2		12	52.2	13	59.1	
>5	1	4.3	1	4.5		0	0	3	13.6	
**Lymph nodes metastasis**										
Negative	17	73.9	1	4.5	**<0.001**	18	78.3	6	27.3	**<0.001**
Positive	6	26.1	21	95.5		5	21.7	16	72.7	
**T staging**										
Tis	4	17.4	0	0	0.22	0	0	3	13.6	0.054
T1	4	17.4	6	27.3		11	47.8	6	27.3	
T2	14	60.9	15	68.2		12	52.2	10	45.5	
T3	1	4.3	1	4.5		0	0	3	13.6	
**pN staging**										
N0	17	73.9	1	4.5	**<0.001**	18	78.3	6	27.3	**0.006**
N1	5	21.7	13	59.1		2	8.7	10	45.5	
N2	1	4.3	6	27.3		1	4.3	3	13.6	
N3	0	0	2	9.1		2	8.7	3	13.6	
**Grade**										
I	1	4.3	1	4.5	0.262	1	4.3	0	0	0.541
II	3	13	8	36.4		12	52.2	13	59.1	
III	10	43.5	7	31.8		8	34.8	6	27.3	
Missing	9	39.1	6	27.3		2	8.7	3	13.6	
**ER**										
Negative	14	60.9	8	36.4	0.094	3	13	3	13.6	0.953
Positive	8	34.8	13	59.1		20	87	19	86.4	
Missing	1	4.3	1	4.5		0	0	0	0	
**PR**										
Negative	14	60.9	8	36.4	0.094	3	13	8	36.4	0.069
Positive	8	34.8	13	59.1		20	87	14	63.6	
Missing	1	4.3	1	4.5		0	0	0	0	
**HER2**										
Negative	15	65.2	19	86.4	0.072	13	56.5	14	63.6	0.626
Positive	7	30.4	2	9.1		10	43.5	8	36.4	
Missing	1	4.3	1	4.5		0	0	0	0	

Furthermore, we collected an additional independent cohort (cohort 2) consisting of 45 pairs of matched BC tissues and adjacent normal tissues. Real‐time qRT‐PCR confirmed that LINC01094 expression was significantly increased in BC tissues compared to adjacent normal tissues (Figure 1D). We also assessed the clinical relevance of LINC01094 in cohort 2. As shown in Table 1, the results were consistent with those from cohort 1, in that LINC01094 expression was significantly higher in the BC tissues of patients with LNM (*n* = 21) compared to those without LNM (*n* = 24) (Figure 1E). Notably, LINC01094 expression exhibited a statistically significant association with pathological nodal (pN) staging. Analyses of ROC curves demonstrated that LINC01094 expression could be used to distinguish patients with LNM from those without LNM, with an AUC of 0.7996 (Figure 1F). These findings reinforced our observations in cohort 1 and supported the link between LINC01094 expression and metastatic progression in BC.

Then, the Coding‐Potential Assessment Tool (CPAT; http://lilab.research.bcm.edu/) and Coding Potential Calculator 2.0 (CPC; http://cpc2.cbi.pku.edu.cn/) predicted that LINC01094 had a low potential for coding proteins (Figure S1C, Supporting information). In addition, RNA pull‐down–western blotting showed that LINC01094 did not bind to ribosomal protein LP0, thereby affirming its weak protein‐coding ability (Figure 1G). Furthermore, we measured LINC01094 expression across a panel of BC cell lines. LINC01094 had high expression in aggressive BC cells (MDA‐MB‐231 and MDA‐MB‐468), but low expression in the non‐tumorigenic human mammary epithelial cell line MCF‐10A (Figure S1D, Supporting information). The subcellular fraction of RNA (Figure 1H) and the fluorescence in situ hybridization (FISH) assays (Figure 1I) revealed that LINC01094 was distributed in the nucleus and cytoplasm, but mainly in the latter. We aimed to assess the stability of LINC01094. A chase assay using an inhibitor of RNA polymerase II, actinomycin D (ActD), was performed. LINC01094 had stable expression in BC cells (Figure 1J).

### 2.2 METTL14‐mediated m^6^A Modification Stabilizes LINC01094 Through IGF2BP2 in BC

**Figure 2:**
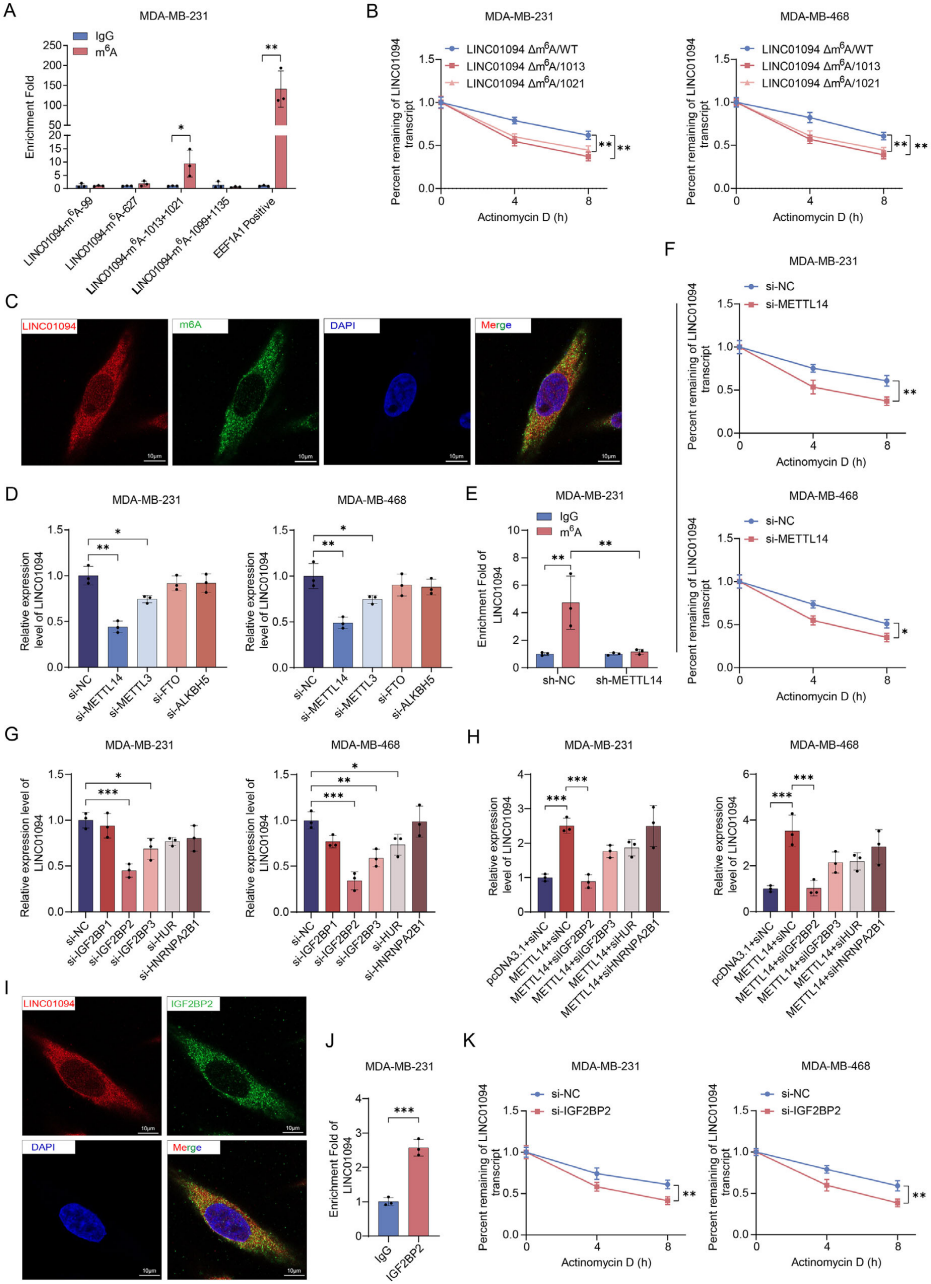
METTL14‐mediated m^6^A modification facilitates the stabilization of LINC01094 through IGF2BP2 in BC. A) The methylated RNA immunoprecipitation (MeRIP) assay was conducted to identify m^6^A modification sites in LINC01094, with EEF1A1 serving as the positive control. B) We conducted ActD chase assays to assess the stability of LINC01094 mutants lacking the m^6^A binding site at position 1013 (△m^6^A/1013) or 1021 (△m^6^A/1021). C) We performed RNA fluorescence in situ hybridization (RNA‐FISH) and immunofluorescence staining, employing fluorescent probes directed at LINC01094 and an antibody designed to specifically bind to m^6^A‐modified RNA molecules in MDA‐MB‐231 cells. The images were captured at an original magnification of ×630. Scale bars: 10 µm. D) The qRT‐PCR assay was employed to assess the impact of writers (METTL3, METTL14) and erasers (FTO, ALKBH5) on the expression of LINC01094 in BC cells. E) MeRIP‐qRT‐PCR assays confirmed that the knockdown of METTL14 led to the inhibition of m^6^A methylation on LINC01094. F) The ActD chase assay confirmed that knocking down METTL14 hindered the stabilization of LINC01094. G) The qRT‐PCR assay was utilized to assess the involvement of m^6^A reader enzymes in the modulation of LINC01094. H) The qRT‐PCR assay confirmed that only the knockdown of IGF2BP2 reversed the increase in LINC01094 induced by METTL14 overexpression. I) We validated the co‐localization of LINC01094 and IGF2BP2 in BC cells through RNA fluorescence in situ hybridization (RNA‐FISH) and immunofluorescence staining in MDA‐MB‐231 cells. The images were captured at an original magnification of ×630. Scale bars: 10 µm. J) LINC01094 exhibited significant enrichment in the fractions immunoprecipitated with IGF2BP2, as demonstrated in the RIP assay. K) The ActD chase assay demonstrated that knocking down IGF2BP2 can inhibit the stability of LINC01094. Data are presented as mean ± SD; statistical significance is indicated (*p < 0.05; **p < 0.01; ***p < 0.001) by Student's t‐test or ANOVA.

Subsequently, we explored the mechanisms underlying the overexpression of LINC01094 in BC. m^6^A is the most abundant post‐transcriptional modification in eukaryotes and plays crucial roles in the epigenetic regulation of lncRNAs. It influences splicing, stability, translation, and other biological functions of RNA.^[^ ^21^ ^]^ To investigate whether the upregulation of LINC01094 expression in BC is mediated by m^6^A modification, we first used SRAMP (www.cuilab.cn/sramp) to predict potential m^6^A‐modification sites in the LINC01094 sequence (Figure S2A,B, Supporting information). Next, we performed a methylated RNA immunoprecipitation (MeRIP) assay based on the predicted m^6^A sites. We identified the 1013+1021 motifs in LINC01094 as the m^6^A modification sites (**Figure** **2**A). We then examined the stability of LINC01094 mutants with deletions of the 1013 m^6^A binding site (△m^6^A/1013) or the 1021 m^6^A binding site (△m^6^A/1021) by conducting the ActD chase assay. Compared to the wild‐type (△m^6^A/WT) group, LINC01094 stability was significantly reduced in the mutants (Figure 2B). Next, we used RNA fluorescence in situ hybridization (RNA‐FISH) and immunofluorescence staining in BC cells with a fluorescent probe targeting LINC01094 and an antibody that binds specifically to m^6^A‐modified RNA molecules. Co‐localization of LINC01094 and m^6^A‐methylated RNA molecules confirmed the presence of m^6^A modification in LINC01094 (Figure 2C; Figure S2C, Supporting information). m^6^A modifies RNA through a dynamic interaction between three homologous factors: “writers” (methyltransferases), “erasers” (demethylases), and “readers” (binding proteins).^[^ ^22^ ^]^ Knockdown of the methyltransferases METTL3 or METTL14, rather than the demethylases FTO or ALKBH5, significantly reduced LINC01094 expression in BC cells (Figure 2D; Figure S2D,E, Supporting information). MeRIP‐qRT‐PCR showed that only knockdown of METTL14 expression led to specific inhibition of m^6^A methylation on LINC01094 (Figure 2E). Moreover, the ActD chase assay confirmed that knockdown of METTL14 expression repressed LINC01094 stability (Figure 2F). In addition, analysis from the GEPIA database (http://gepia.cancer‐pku.cn/) revealed a positive correlation between METTL14 and LINC01094 expression (Figure S2F, Supporting information). These results indicate that METTL14‐mediated m^6^A modification contributes to the upregulation of LINC01094 expression in BC.

The biological significance of m^6^A modification depends on m^6^A reader proteins, which specifically decode m^6^A mark sites and mediate the stability of methylated RNAs.^[^ ^23^ ^]^ We aimed to identify the reader protein that recognizes LINC01094. We silenced the expression of several m^6^A reader proteins (IGF2BP1, IGF2BP2, IGF2BP3, HUR, and HNRNPA2B1) and measured LINC01094 expression (Figure S2G,H, Supporting information). Knockdown of IGF2BP2 and IGF2BP3 significantly reduced LINC01094 expression in BC cells (Figure 2G). However, silencing IGF2BP2 expression was sufficient to reverse the enhanced LINC01094 expression induced by METTL14 overexpression (Figure 2H). Additionally, RNA‐FISH and immunofluorescence staining confirmed co‐localization of LINC01094 and IGF2BP2 in BC cells (Figure 2I; Figure S2I, Supporting information). Consistently, LINC01094 expression was significantly enriched in IGF2BP2‐immunoprecipitated fractions, as shown in the RNA immunoprecipitation (RIP) assay (Figure 2J), suggesting a strong interaction between IGF2BP2 and LINC01094. Moreover, analysis from the GEPIA database revealed a positive correlation between IGF2BP2 protein levels and LINC01094 expression in BC patients (Figure S2J, Supporting information). These data confirm that IGF2BP2 is a major m^6^A reader that recognizes METTL14‐mediated methylated LINC01094. IGF2BP2 is essential for RNA stability in an m^6^A‐dependent manner.^[^ ^24^ ^]^ We further explored the effect of m^6^A modification on LINC01094 stability. The ActD chase assay showed that the half‐life of LINC01094 in the IGF2BP2‐silencing group was significantly shorter than in the control group after ActD treatment (Figure 2K). These findings suggest that METTL14‐mediated m^6^A modification upregulates LINC01094 expression through an IGF2BP2‐dependent stabilization mechanism.

### 2.3 LINC01094 Interacts with PKM2 and Facilitates PKM2 Dimeric Assembly

**Figure 3:**
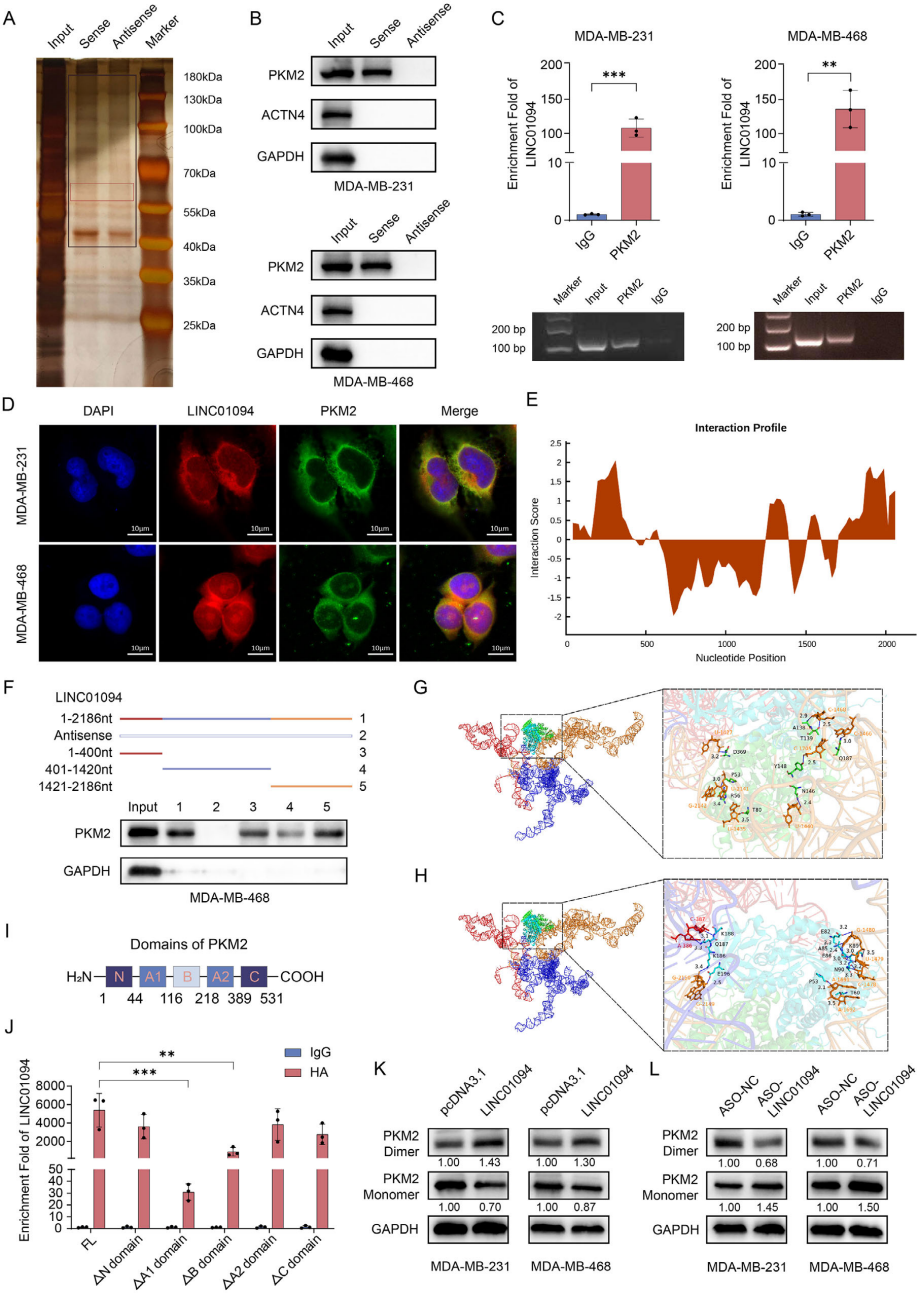
LINC01094 interacts with PKM2, promoting its dimeric assembly. A) Silver staining following the RNA pull‐down assay identified proteins pulled down by LINC01094 in MDA‐MB‐231 cells, with a red box indicating the differential band corresponding to PKM2. B) RNA pull‐down‐western blot assays validated the specific interaction between PKM2 and LINC01094 in BC cells. C) RIP assay confirmed the association of PKM2 with LINC01094. D) RNA‐FISH and immunofluorescence staining confirmed the co‐localization of LINC01094 and PKM2 in BC cells, with images captured at ×630 original magnification. Scale bars: 10 µm. E) The catRAPID website predicted the existence of binding sites between LINC01094 and PKM2. F) RNA pull‐down combined with western blot assays verified the binding regions of LINC01094 and PKM2. G, H) Schematic diagrams illustrating the hydrogen bond interaction between the protein A chain (labeled as green) (G) or B chain (labeled as cyan) (H) of the PKM2 dimer and LINC01094. I) Schematic of PKM2 structural domains. Deletions of specific domains (ΔN, ΔA1, ΔB, ΔA2, and ΔC) were generated. J) RIP assay validation of LINC01094 binding. RNA immunoprecipitation showed that ΔA1 and ΔB mutants exhibited significantly reduced LINC01094 enrichment compared to WT PKM2. K, L) Following cross‐linking with disuccinimidyl suberate (DSS), cellular lysates were subjected to western blot analysis to assess the impact of LINC01094 expression on the abundance of PKM2 monomers and dimers. Data are presented as mean ± SD; statistical significance is indicated (**p < 0.01; ***p < 0.001) by Student's t‐test or ANOVA.

LncRNAs regulate genes primarily by binding to RNA‐binding proteins (RBPs) or absorbing miRNAs as competing endogenous RNAs (ceRNAs).^[^ ^25^ ^]^ We aimed to dissect the mechanistic role of LINC01094 in carcinogenesis. We applied an RNA pull‐down assay in combination with silver‐staining sodium dodecyl sulfate–polyacrylamide gel electrophoresis to screen for lncRNA‐interacting proteins. A specific band at 40–180 kDa, pulled down by LINC01094 sense and antisense control RNA, was sent to a mass spectrometer to identify the potential proteins captured by LINC01094 (**Figure** **3**A). Mass spectrometry revealed that argonaute 2 (AGO2) protein, the key component of RNA‐induced silencing complexes, was not enriched by LINC01094, indicating that LINC01094 did not regulate BC progression as a ceRNA. Next, we screened for entities that are physically bound to LINC01094. PKM2 and ACTN4 stood out among the candidates, exhibiting higher scores within the range of 40–180 kDa. We then performed RNA pull‐down–western blotting and verified that only PKM2 was bound specifically to LINC01094 in BC cells (Figure 3B). The RIP assay further validated the association between LINC01094 and PKM2 (Figure 3C). In addition, RNA‐FISH and immunofluorescence staining demonstrated the co‐localization of LINC01094 and PKM2 in BC cells (Figure 3D). Furthermore, we used catRAPID (http://service.tartaglialab.com/page/catrapid_group) to analyze the potential binding sites between LINC01094 and PKM2 (Figure 3E).

To identify the binding sites precisely, we generated and selected the optimal 3D model of LINC01094 (Figure S3A, Supporting information). Based on the predicted structure and functional domains of LINC01094, we constructed three biotinylated truncations (A: 1–400‐nt; B: 401–1420‐nt; C: 1421–2186‐nt) of LINC01094, followed by RNA pull‐down–western blotting. The regions of 1–400‐nt and 1421–2186‐nt were identified as the main binding sequences of LINC01094 with PKM2 monomers (Figure 3F). Interestingly, the two regions in LINC01094 that were bound to PKM2 were physically close in space. Hence, we hypothesized that LINC01094 promoted PKM2 dimeric assembly from monomers as a “molecular scaffold.” We then conducted nucleic acid–protein molecular docking of PKM2 and LINC01094 using the HDOCK tool and selected configurations with the highest docking and confidence scores for further analyses (Figure S3B, Supporting information). Structural analyses revealed that the protein A chain of the PKM2 dimer (labeled green) was bound specifically to the 1421–2186‐nt stem‐loop sequence of LINC01094 (orange) through hydrogen‐bond interactions (Figure 3G; Figure S3C, Supporting information). The B chain of the PKM2 dimer (cyan) demonstrated binding affinity for the 1–400‐nt stem‐loop sequence of LINC01094 (red) (Figure 3H; Figure S3D, Supporting information). We then undertook a series of truncation experiments based on the predicted binding interface and known PKM2 structural domains.^[^ ^26^ ^]^ Specifically, we generated a panel of PKM2 mutants by deleting distinct domains: ΔN, ΔA1, ΔB, ΔA2, and ΔC (Figure 3I). We then carried out RIP experiments with these truncated constructs to assess their ability to pull down LINC01094. Deletion of the A1 and B domains reduced the interaction with LINC01094 significantly, whereas mutations in other domains did not have such a marked effect (Figure 3J, Figure S3E, Supporting information). These findings directly supported the predicted molecular‐docking model and confirmed that the A1 and B domains were critical for the binding of PKM2 to LINC01094. Disuccinimidyl suberate (DSS) crosslinking–western blotting showed that upregulation of LINC01094 expression reduced the abundance of PKM2 monomers but increased its dimeric forms (Figure 3K). Knockdown of LINC01094 expression promoted the monomeric form of PKM2 but hindered its dimeric form (Figure 3L). Subsequently, we transfected full‐length LINC01094 and its truncated variants into BC cells for DSS crosslinking–western blotting. Compared with the full‐length group, the truncated variant lacking the binding sequence with PKM2 did not exhibit any effect in promoting the formation of the PKM2 dimer (Figure S3F, Supporting information). Taken together, these data indicated that LINC01094 could bind directly with PKM2 and promote PKM2 dimeric assembly.

### 2.4 LINC01094 Facilitates the Formation of PKM2/JMJD5 Complexes and Promotes the Nuclear Translocation of PKM2

**Figure 4:**
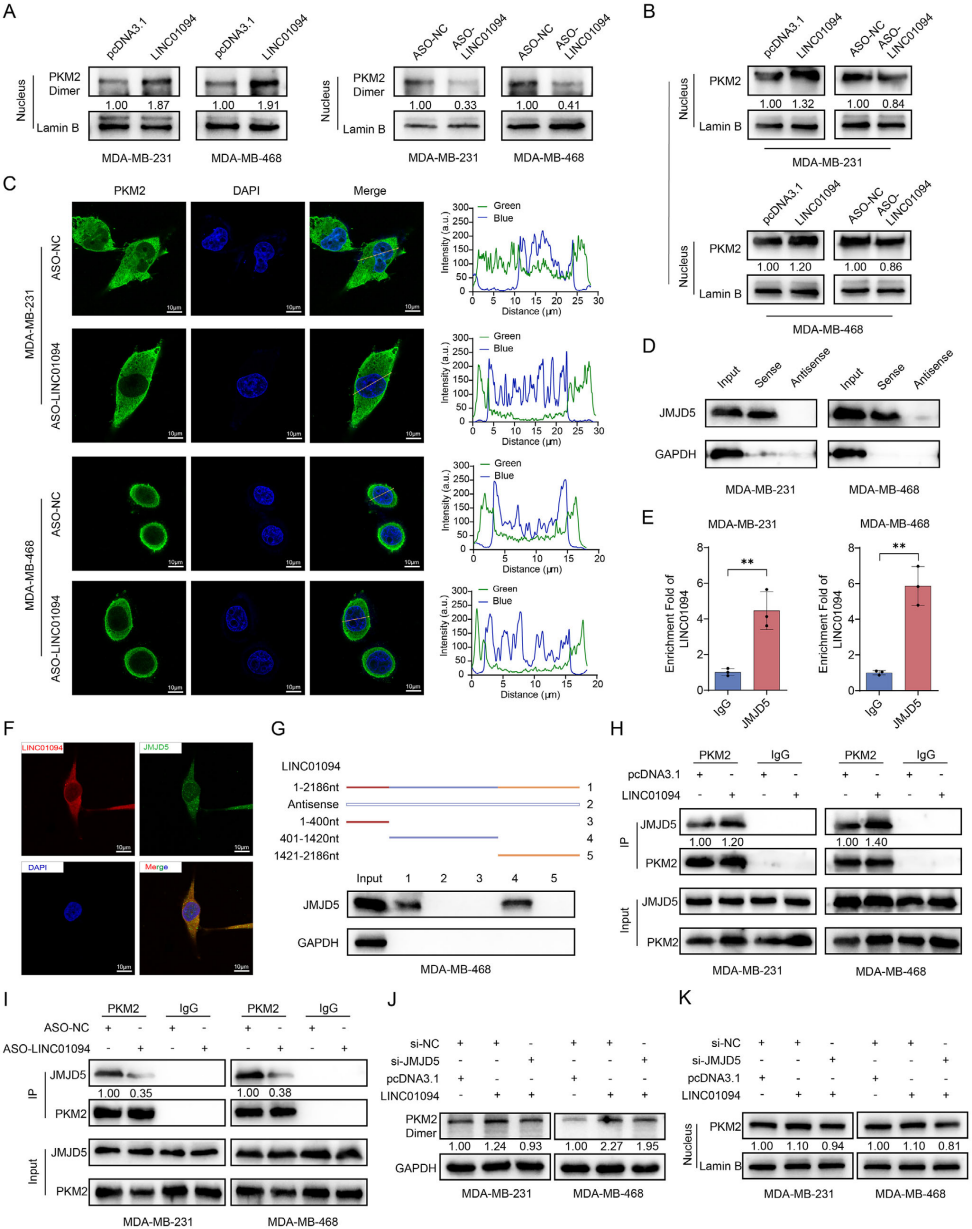
LINC01094 facilitates the formation of PKM2/JMJD5 complexes and enhances the nuclear translocation of PKM2. A) Cross‐linking‐western blot assays detected the impact of LINC01094 on the nuclear distribution of dimeric PKM2. B) Western blot analysis assessed the influence of LINC01094 on the nuclear distribution of PKM2. C) Immunofluorescence staining confirmed that repressing the expression of LINC01094 can significantly inhibit the nuclear distribution of PKM2. The images were captured at an original magnification of ×630. Scale bars: 10 µm. D) RNA pull‐down‐western blot assays revealed that JMJD5 could be pulled down by LINC01094 sense, using its antisense as the negative control. E) The RIP assay revealed a significant enrichment of LINC01094 in the pull‐down products of JMJD5. F) Combined RNA‐FISH and immunofluorescence staining unequivocally confirmed the co‐localization of LINC01094 and JMJD5 within MDA‐MB‐231 cells. The images were captured using an original magnification of ×630. Scale bars: 10 µm. G) RNA pull‐down assays revealed that the 401–1420 nt segment of LINC01094 interacts with JMJD5. H, I) Co‐IP assays verified that overexpression (H) or knockdown (I) of LINC01094 could respectively enhance or inhibit the binding ability of PKM2 and JMJD5. J) The DSS crosslinking‐western blot assays showed that LINC01094 could enhance PKM2 dimerization, but this impact was reversed upon JMJD5 knockdown. K) The western blot assays demonstrated that LINC01094 increased the expression of nuclear PKM2, but this effect was reversed by JMJD5 knockdown. Data are presented as mean ± SD; statistical significance is indicated (**p < 0.01) by Student's t‐test.

The PKM2 dimer has been shown to translocate into the nucleus and act as a transcriptional coactivator or phosphotransferase to modulate the transcriptional program.^[^ ^27^ ^]^ Therefore, we further investigated the influence of LINC01094 on the nuclear translocation of PKM2. DSS crosslinking‐western blotting revealed that an increase in LINC01094 expression enhanced the presence of dimeric PKM2 in the nucleus, while its deletion led to a parallel reduction (**Figure** **4**A). Next, western blotting showed that LINC01094 overexpression significantly increased PKM2 distribution in the nucleus, whereas LINC01094 knockdown led to the opposite trend (Figure 4B). PKM2 distribution was visualized using confocal microscopy after RNA‐FISH and immunofluorescence staining. In control cells, PKM2 was predominantly located in the cytoplasm, and LINC01094‐deficient cells retained a lower level of nuclear PKM2 compared to the negative‐control group (Figure 4C). However, upregulation of LINC01094 expression promoted the nuclear translocation of PKM2 (Figure S4A, Supporting information). We next explored the role of LINC01094 in facilitating the nuclear translocation of PKM2. Previous studies have shown that JMJD5 hinders PKM2 tetramerization and promotes the nuclear distribution of PKM2 by physically binding to it.^[^ ^28^ ^]^ Thus, we hypothesized that LINC01094 may mediate the formation of PKM2/JMJD5 complexes by acting as a molecular scaffold, thereby facilitating the conversion of PKM2 from a tetramer to a dimer and enhancing its translocation into the nucleus. To test this hypothesis, RNA pull‐down‐western blotting showed that JMJD5 was pulled down from BC cell lysates by in vitro transcribed biotinylated LINC01094, but not by antisense LINC01094 (Figure 4D). Furthermore, in the RIP assay, LINC01094 was enriched with anti‐JMJD5 antibodies but not in IgG immunoprecipitates (Figure 4E; Figure S4B, Supporting information). Additionally, RNA‐FISH and immunofluorescence staining confirmed the co‐localization of LINC01094 and JMJD5 in BC cells (Figure 4F; Figure S4C, Supporting information).

To better understand the interaction between LINC01094 and the PKM2/JMJD5 complex, we carried out RNA pull‐down assays using truncated fragments of LINC01094. These experiments revealed that the 401‐1420‐nt segment of LINC01094 interacted with JMJD5 (Figure 4G). Our previous results showed that LINC01094 could bind to both PKM2 and JMJD5. We then investigated whether LINC01094 mediates binding between PKM2 and JMJD5 by serving as a “flexible scaffold.” Co‐immunoprecipitation (Co‐IP) assays demonstrated that PKM2 and JMJD5 bound to each other in BC cells (Figure S4D, Supporting information). This interaction was further promoted by LINC01094 overexpression (Figure 4H), whereas knockdown of LINC01094 expression significantly reduced the PKM2/JMJD5 interaction (Figure 4I). Additional experiments revealed that LINC01094 promoted the dimerization of PKM2, an effect that was reversed by knockdown of JMJD5 expression (Figure 4J). Similarly, LINC01094 enhanced the nuclear expression of PKM2, which was also reversible upon knockdown of JMJD5 expression (Figure 4K). These results suggest that LINC01094 facilitates the dimerization of PKM2 and its nuclear translocation by interacting with JMJD5. Collectively, these findings demonstrate that LINC01094 promotes the formation of PKM2/JMJD5 complexes by serving as a scaffold and enhances the translocation of PKM2 into the nucleus.

### 2.5 LINC01094 Promotes the Interaction of PKM2 with HIF‐1α and β‐Catenin and Facilitates Their Transactivation Ability

**Figure 5:**
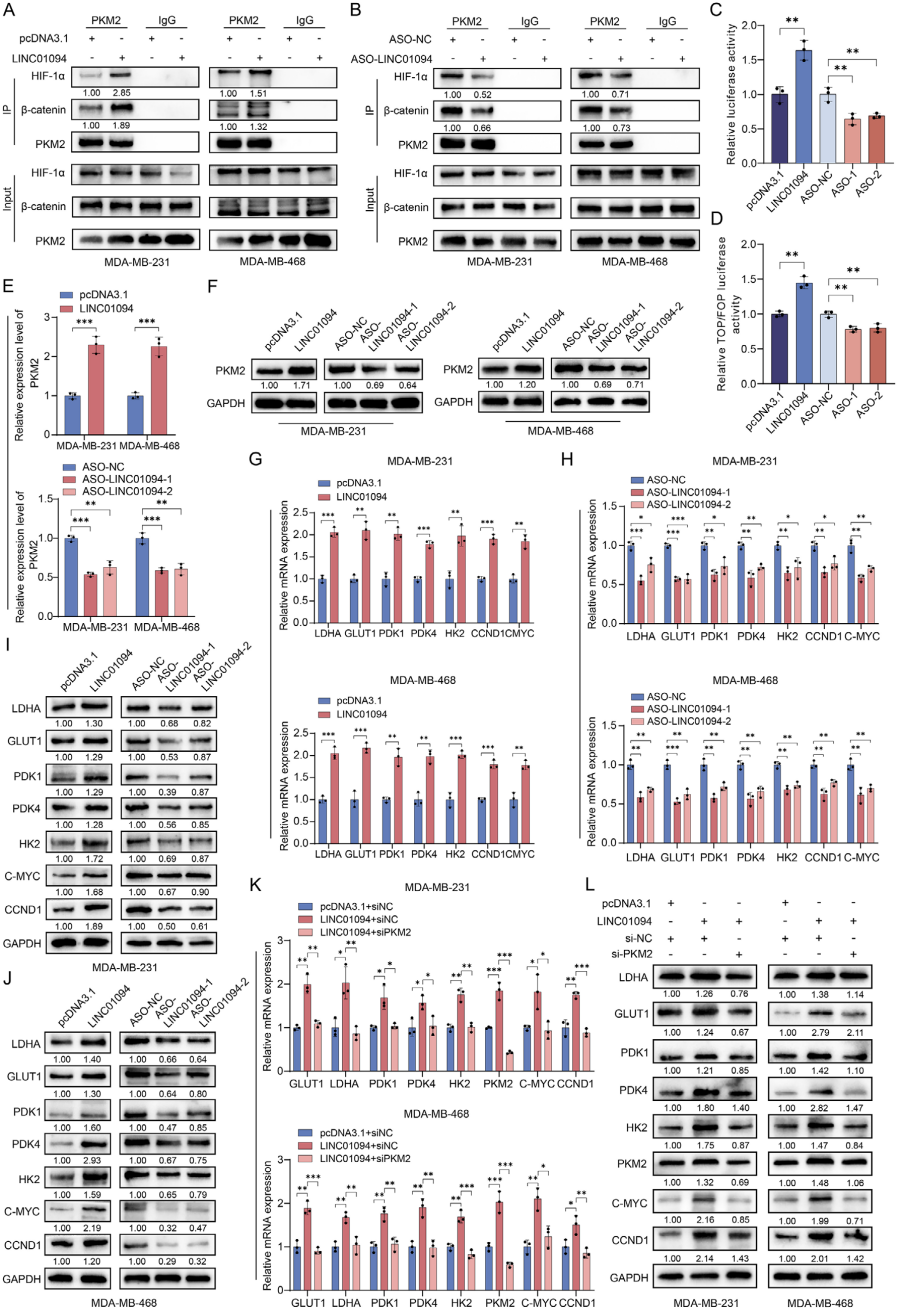
LINC01094 enhances the interaction between PKM2, HIF1‐α, and β‐catenin, promoting their transactivation activity. A, B) Co‐IP assays showed that LINC01094 overexpression enhanced (A), while knockdown inhibited (B), the binding of PKM2 and HIF‐1α, or β‐catenin. C, D) Dual‐luciferase reporter assays demonstrated that LINC01094 overexpression enhanced the transcriptional activities of both HIF1‐α (C) and β‐catenin (D), while knockdown of LINC01094 reduced these effects. E, F) Elevating LINC01094 in BC cells directly enhanced PKM2, while its deletion significantly reduced PKM2 levels, as indicated by the experimental results for qRT‐PCR (E) and western blot assays (F). G‐L) qRT‐PCR (G, H, and K) and western blot assays (I, J, and L) confirmed that LINC01094 enhanced the expression of energy metabolism enzymes through PKM2. Data are presented as mean ± SD; statistical significance is indicated (*p < 0.05; **p < 0.01; ***p < 0.001) by Student's t‐test or ANOVA.

PKM2, when translocated into the nucleus, functions as a transcriptional coactivator for HIF‐1α, β‐catenin, and Stat3, accelerating carcinogenesis.^[^ ^29^ ^]^ Co‐IP assays revealed that upregulation of LINC01094 expression significantly promoted the interaction between PKM2 and both HIF‐1α and β‐catenin (**Figure** **5**A). In contrast, knockdown of LINC01094 expression inhibited the interaction between PKM2 and these two proteins (Figure 5B). To assess the functional consequences of these interactions, we performed luciferase reporter assays to investigate the role of LINC01094 in enhancing the transactivation ability of HIF‐1α and β‐catenin. For HIF‐1α, we used a luciferase reporter vector containing hypoxia‐responsive elements (HREs), which are bound by HIF‐1α to activate transcription. For β‐catenin, we employed the TOPflash reporter plasmid containing TCF/LEF binding sites targeted by the β‐catenin/TCF complex, with the FOPflash reporter plasmid (containing mutated TCF/LEF sites) serving as a negative control. LINC01094 overexpression significantly enhanced the transactivation ability of both HIF‐1α and β‐catenin, whereas knockdown of LINC01094 expression suppressed this ability (Figure 5C,D). These results provide direct functional evidence that LINC01094 modulates HIF‐1α‐ and β‐catenin‐driven transcriptional programs.

Previous studies have reported that PKM2 promotes the transactivation of HIF‐1α‐targeted genes (including PKM2 itself), thereby forming a positive feedback loop that triggers a cascade reaction in aerobic glycolysis and cancer progression.^[^ ^30^ ^]^ Consistently, increased expression of LINC01094 enhanced PKM2 levels in BC cells, while its deletion led to a significant reduction in PKM2 expression (Figure 5E,F). Moreover, real‐time qRT‐PCR analysis showed that LINC01094 overexpression resulted in a marked increase in HIF‐1α‐targeted genes, including LDHA, GLUT1, PDK1, PDK4, and HK2, as well as genes regulated by β‐catenin, such as CCND1 and CMYC (Figure 5G). Knockdown of LINC01094 expression showed the opposite effect (Figure 5H). These findings were also confirmed at the protein level (Figure 5I,J). Database analyses indicated a significant positive correlation between LINC01094 and the HIF‐1α‐targeted genes (Figure S5A,B, Supporting information). Consistently, knockdown of PKM2 expression reversed the stimulatory effect of LINC01094 on the downstream targets of HIF‐1α and β‐catenin at both the RNA and protein levels (Figure 5K,L). Furthermore, knockdown of HIF‐1α expression could reverse the promoting effect of LINC01094 on the downstream regulatory genes of HIF‐1α (Figure S5C,D, Supporting information). In summary, our data uncover that LINC01094 acts as a “molecular chaperone” for PKM2, facilitating its interaction with HIF‐1α and β‐catenin and further enhancing the transactivation of HIF‐1α and β‐catenin target genes.

### 2.6 LINC01094 Promotes Aerobic Glycolysis and Tumor Progression in BC

**Figure 6:**
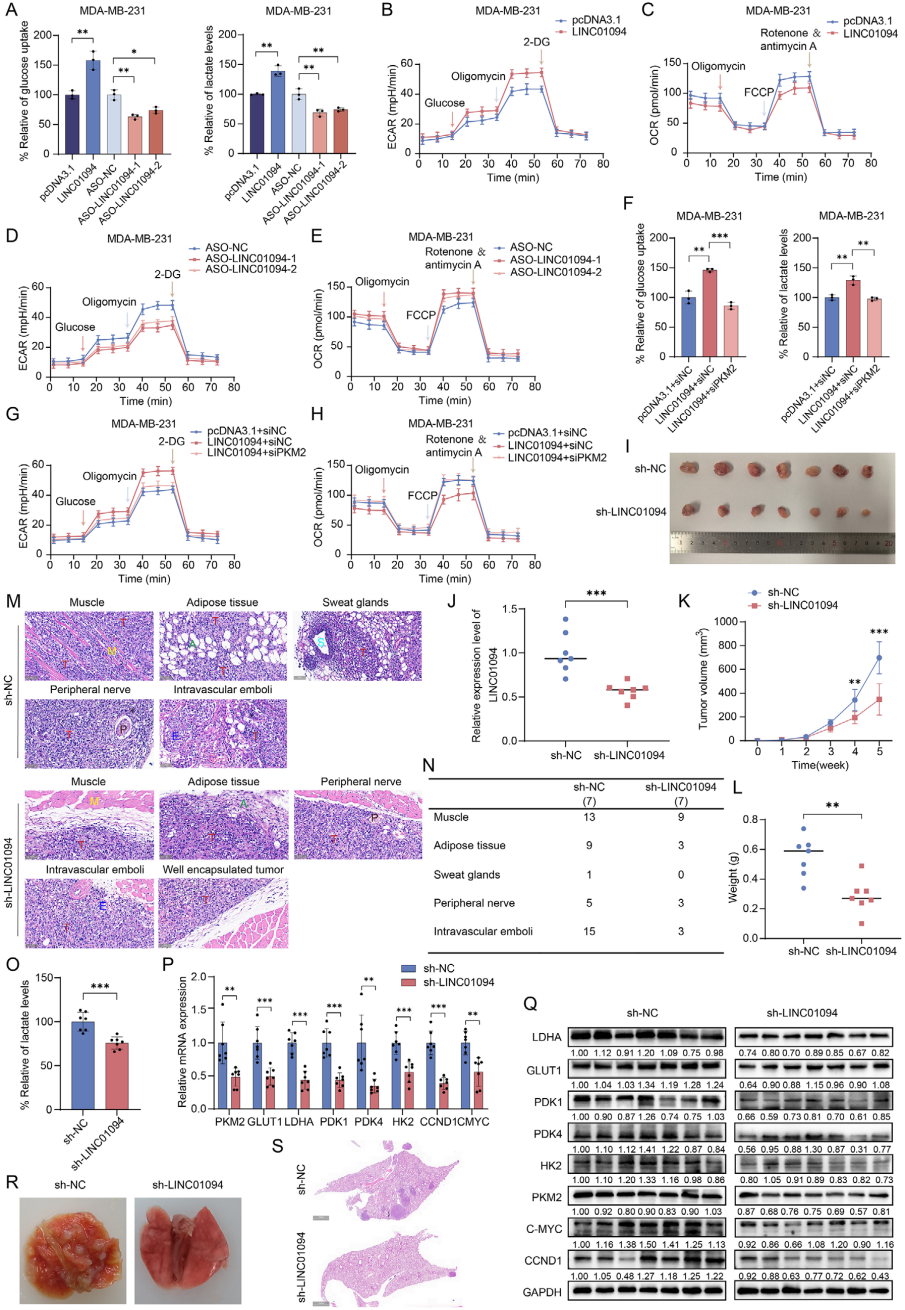
LINC01094 promotes aerobic glycolysis and contributes to tumor progression in BC. A‐H) The levels of glucose uptake, lactate production (A, F), ECAR (B, D, and G), and OCR (C, E, and H) were measured. The results indicated that LINC01094 could promote the energy metabolism reprogramming of BC cells through PKM2. I) The subcutaneous xenografts in mice were excised and photographed. J) qRT‐PCR assay was used to detect the expression of LINC01094 in the subcutaneous tumors extracted from two separate groups of mice. K, L) The growth (K) and weight (L) patterns of the xenograft tumors were measured. M) H&E staining of xenograft tumors showed that the LV‐sh LINC01094 and LV‐NC groups had local infiltration into nearby interstitial and peripheral nerve tissues, forming intravascular emboli. In contrast, tumors injected with LV‐sh LINC01094 exhibited better encapsulation compared to the LV‐NC group. Scale bars: 50 µm. N) The table summarizes the total number of cancerous lesions for each infiltration pattern in each mouse group. O) Lactate levels were assessed in tumors extracted from each mouse. P, Q) qRT‐PCR (P) and western blot assays (Q) were employed to identify genes related to energy metabolism in subcutaneous tumors obtained from each mouse. (R) Photographs of the lungs removed from the hematogenous metastasis model of mice. (S) Microscopic images of H&E‐stained lung sections from mice. Scale bars: 1000 µm. Data are presented as mean ± SD; statistical significance is indicated (*p < 0.05; **p < 0.01; ***p < 0.001) by Student's t‐test or ANOVA.

LINC01094 promotes aerobic glycolysis and tumor progression in BC. PKM2 is a critical glycolytic enzyme. We verified that LINC01094 promotes the dimeric assembly of PKM2 and enhances its translocation into the nucleus by forming PKM2/JMJD5 complexes, thereby facilitating the transactivation activity of aerobic glycolysis‐related genes induced by HIF‐1α. Hence, we investigated whether LINC01094 plays a critical role in glycolytic metabolism in BC. To test this hypothesis, we first assessed the dose‐dependent effects of LINC01094, ranging from 0 to 3 µg. We found that 2 µg produced an optimal effect on tumor energy metabolism, with no further enhancement observed at 3 µg (Figure S6A,B, Supporting information). Therefore, 2 µg was used in subsequent experiments. To evaluate the long‐term impact, we overexpressed LINC01094 in BC cells and monitored its effects over 0, 6, 12, 24, 48, and 72 h. LINC01094 significantly increased cellular metabolism starting at 24 h, stabilizing by 48 h (Figure S6C,D, Supporting information). As expected, we found that LINC01094‐overexpressing cells showed a significant increase in glucose uptake and lactate production, whereas LINC01094‐knockdown cells exhibited the opposite metabolic flux (**Figure** **6**A; Figure S6E‐G, Supporting information). Moreover, increased LINC01094 expression enhanced the extracellular acidification rate (ECAR, indicative of increased glycolytic flux) but inhibited the oxygen consumption rate (OCR, reflecting decreased mitochondrial respiratory capacity) (Figure 6B,C; Figure S6H,I, Supporting information). Consistently, downregulation of LINC01094 expression suppressed ECAR and boosted OCR in BC cells (Figure 6D,E; Figure S6J,K, Supporting information). Furthermore, the augmentation of glucose uptake, lactate production, ECAR, and the reduction of OCR conferred by LINC01094 overexpression could be counteracted by inhibiting PKM2 expression in BC cells (Figure 6F‐H; Figure S6L‐N, Supporting information). These data reveal that LINC01094 promotes aerobic glycolysis in BC cells through PKM2. Reprogrammed patterns of glycolytic metabolism, a hallmark of malignancy, facilitate cancer progression.^[^ ^31^ ^]^ We further determined the optimal concentration of LINC01094 for its effects on BC progression. We discovered that 2 µg produced the strongest response, with no further enhancement at higher doses (Figure S7A,C, Supporting information). This concentration was used in subsequent assays. To evaluate the long‐term impact, we overexpressed LINC01094 in BC cells and monitored its effects over 0, 6, 12, 24, 48, and 72 h. LINC01094 significantly increased tumor progression starting at 24 h, with effects stabilizing by 48 h (Figure S7B,D, Supporting information). Consistently, CCK8 (Figure S8A,B, Supporting information), EdU (Figure S8C,D, Supporting information), and Transwell assays (Figure S8E‐H, Supporting information) verified that LINC01094 significantly promoted the proliferation, migration, and invasion capabilities of BC cells.

We aimed to investigate the role of LINC01094 in PKM2‐mediated metabolic reprogramming and tumor progression. We undertook comprehensive functional assays, including glucose uptake, lactate production, cell proliferation (CCK‐8 assay), and migration/invasion (Transwell assays). PKM2 overexpression enhanced glucose metabolism (glucose uptake and lactate production) significantly (Figure S9A,B, Supporting information) and promoted tumor progression (proliferation, migration, and invasion) significantly (Figure S9C‐E, Supporting information). However, when PKM2 was overexpressed in LINC01094‐knockdown cells, these effects were markedly attenuated (Figure S9A‐E, Supporting information), indicating that LINC01094 is crucial for PKM2‐mediated metabolic reprogramming and oncogenic functions in BC cells. To further explore the interplay between LINC01094 and PKM2, we co‐overexpressed both molecules in BC cells. PKM2 overexpression enhanced metabolic reprogramming and tumor progression. Notably, co‐overexpression of LINC01094 with PKM2 further amplified these effects compared to PKM2 overexpression alone (Figure S9F‐J, Supporting information). These findings underscore the essential role of LINC01094 in the metabolic and oncogenic activity of PKM2 in BC cells, with its overexpression further potentiating the effects of PKM2.

Next, we established a xenograft tumor model by subcutaneously implanting BC cells transfected with GFP‐expressed lentiviruses LV‐sh LINC01094 or LV‐sh NC into the mammary fat pad of mice to corroborate the role of LINC01094 in regulating BC progression in vivo (Figure 6I). The xenograft tumors harvested from the LV‐sh LINC01094 group exhibited significantly lower expression of LINC01094 compared to that in the LV‐sh NC group (Figure 6J). Tumor growth in the LINC01094‐downregulated group was inhibited markedly compared to that in the negative control group (Figure 6K,L). Afterward, we conducted statistical analyses of peritumor infiltration in xenografted mice, encompassing islands of cancer cells infiltrating stromal tissues (e.g., muscle, adipose tissue, and sweat glands), as well as peripheral nerves and intravascular emboli (Figure 6M). The total number of cancerous lesions corresponding to each infiltration pattern in each group is summarized in Figure 6N. Silencing of LINC01094 expression significantly curtailed the local infiltration capability of xenografts, with tumor nodules in the LV‐sh LINC01094 group exhibiting improved encapsulation. Interestingly, the lactate production measured in the tumors resected from the LV‐sh LINC01094 group was markedly restrained compared with that derived from the control group (Figure 6O). Furthermore, real‐time qRT‐PCR and western blotting confirmed that downregulation of LINC01094 expression inhibited the expression of HIF‐1α (PKM2, LDHA, GLUT1, PDK1, PDK4, and HK2)‐ and β‐catenin (CCND1 and CMYC)‐targeted genes significantly (Figure 6P,Q). To assess the impact of LINC01094 on invasion and metastasis, we established a hematogenous metastasis model by injecting BC cells transfected with LV‐sh LINC01094 or LV‐sh NC into the tail vein of mice. Fewer and smaller metastatic loci were detected by in vivo imaging and hematoxylin‐and‐eosin staining in the LV‐sh LINC01094 group compared to the LV‐sh NC group (Figure 6R,S), indicating that knockdown of LINC01094 expression suppressed the lung metastasis of BC. Taken together, these data suggest that LINC01094 promotes glycolytic metabolic reprogramming and tumor progression in BC both in vitro and in vivo.

### 2.7 LINC01094 is a Potential Therapeutic Target for BC

**Figure 7:**
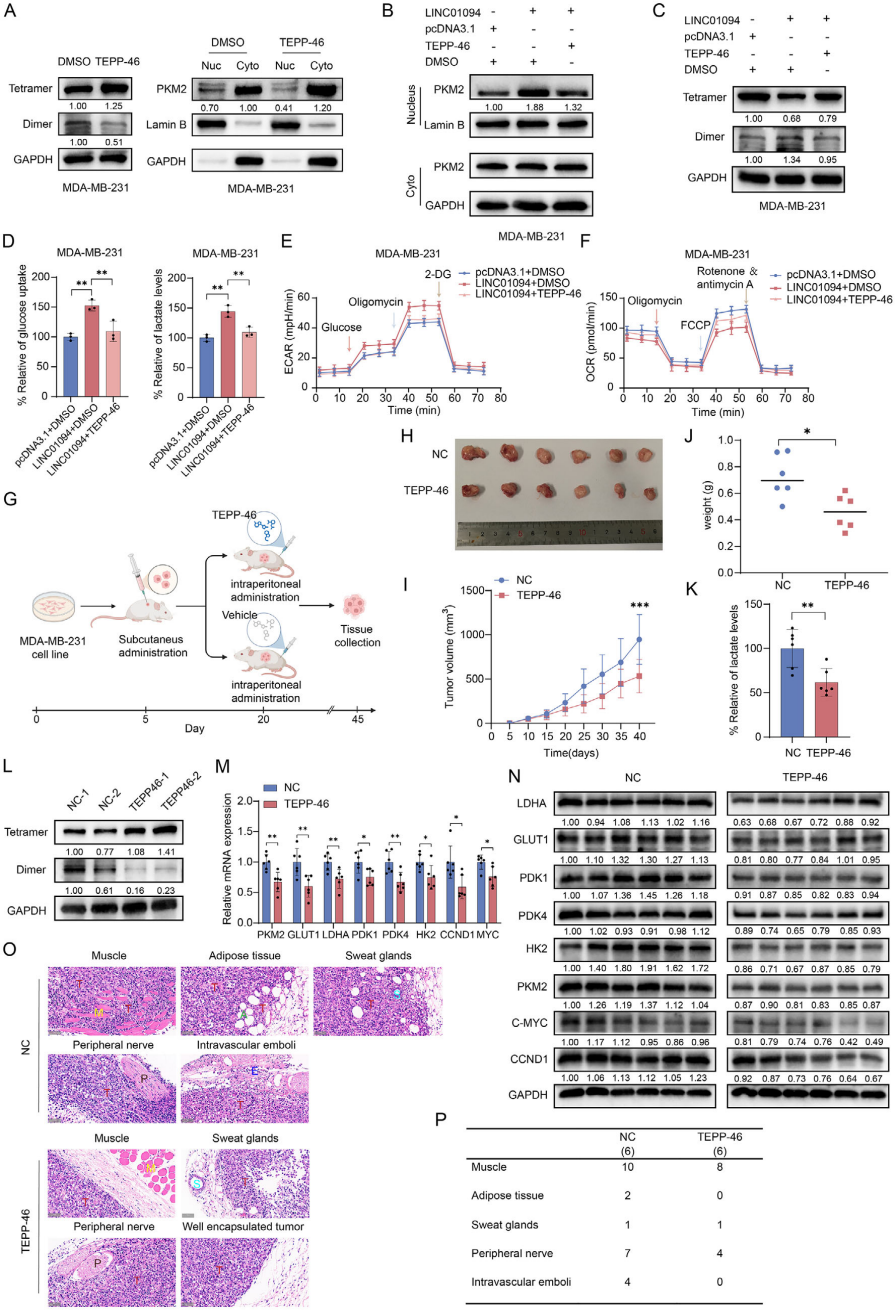
LINC01094 is a promising therapeutic target for BC. A) Western blot assays following DSS crosslinking revealed a notable reduction in dimeric PKM2 forms and nuclear PKM2 upon TEPP‐46 treatment in BC cells. B) Intracellular analysis showed that LINC01094 up‐regulation increased PKM2 distribution in the nucleus, but this effect was significantly reversed by TEPP‐46 treatment. C) Western blot assays following DSS crosslinking demonstrated that TEPP‐46 effectively nullified the enhancement of PKM2 dimerization induced by LINC01094. D‐F) Glucose uptake, lactate production (D), ECAR (E), and OCR (F) assays confirmed that TEPP‐46 could reverse the promotion of energy metabolism reprogramming induced by LINC01094 overexpression in MDA‐MB‐231 cells. G) The workflow diagram for in vivo experiments in mice. H) The subcutaneous xenografts were surgically removed and photographed. I, J) The growth (I) and weight (J) of the xenograft tumors were assessed and recorded. K) Lactate levels were measured in tumors obtained from each mouse. L) DSS crosslinking‐western blot analysis of tumors verified that TEPP‐46 effectively inhibited the formation of PKM2 dimers. M, N) Genes related to energy metabolism in subcutaneous tumors from each mouse were identified using qRT‐PCR (M) and western blot assays (N). O, P) The xenograft tumors were visually represented through H&E staining (O), accompanied by a statistical table indicating incidence rates for each infiltration condition (P). Scale bars: 50 µm. Data are presented as mean ± SD; statistical significance is indicated (*p < 0.05; **p < 0.01; ***p < 0.001) by Student's t‐test or ANOVA.

TEPP‐46 is a small‐molecule activator that inhibits the dimeric form of PKM2.^[^ ^32^ ^]^ Thus, TEPP‐46 could be used as a functional blocker of LINC01094. First, we assessed the impact of TEPP‐46 on PKM2 in vitro. DSS crosslinking‐western blotting showed that the abundance of dimer forms of PKM2 decreased significantly, and the nuclear translocation of PKM2 was hindered after treatment with TEPP‐46 in the LINC01094‐highly‐expressed MDA‐MB‐231 cells (**Figure** **7**A). Moreover, intracellular distribution analyses revealed that TEPP‐46 markedly reversed the increase in PKM2 distribution in the nucleus induced by upregulation of LINC01094 expression (Figure 7B; Figure S10A, Supporting information). In addition, the increase in the abundance of PKM2 dimers promoted by LINC01094 could be eliminated by TEPP‐46 (Figure 7C; Figure S10B, Supporting information). Next, we investigated whether TEPP‐46 could reverse the effect of LINC01094 on energy metabolism and tumor progression in BC. First, we determined the optimal concentration and time effects of TEPP‐46 on the energy metabolism and tumor progression of BC cells. Gradient‐concentration tests identified 50 µM as the optimal dose, as further increases in concentration did not enhance its inhibitory effects (Figure S10C,D,G, Supporting information). Then, we assessed the long‐term effects of TEPP‐46 by treating BC cells and analyzing the endpoints at 0, 6, 12, 24, 48, and 72 h. TEPP‐46 began to suppress energy metabolism and tumor progression in BC cells at 24 h, with the effects becoming more pronounced and stabilizing by 48 h (Figure S10E,F,H, Supporting information). As expected, TEPP‐46 could reverse the promotion of energy metabolism reprogramming and upregulation of downstream genes induced by LINC01094 overexpression in BC cells (Figure 7D‐F; Figure S10I‐L, Supporting information).

Next, we evaluated whether LINC01094 could serve as a potential therapeutic target for BC in vivo. We established a xenograft tumor model by subcutaneously implanting MDA‐MB‐231 cells into the mammary fat pad of nude mice. Then, TEPP‐46 was injected (i.p.) into the mice daily when the subcutaneous tumor volume reached 125 mm^3^ (Figure 7G). Mice treated with TEPP‐46 exhibited a significant decrease in tumor burden (Figure 7H‐J) and lactate production (Figure 7K) compared to the control group. DSS crosslinking–western blotting showed that TEPP‐46 inhibited the formation of PKM2 dimers in the xenotransplanted tumors resected from mice (Figure 7L). Moreover, real‐time qRT‐PCR and western blotting revealed that TEPP‐46 suppressed the expression of HIF‐1α (PKM2, LDHA, GLUT1, PDK1, PDK4, and HK2)‐ and β‐catenin (CCND1 and CMYC)‐targeted genes markedly (Figure 7M,N). Subsequently, we conducted statistical analyses on peritumoral infiltration in xenografted mice, observing cancer‐cell clusters infiltrating stromal tissues, peripheral nerves, and intravascular emboli (Figure 7O). Detailed statistical analyses are presented in Figure 7P. TEPP‐46 injection significantly inhibited the local infiltration capability of xenografts, and the tumor nodules in the TEPP‐46‐injection group were better encapsulated. Taken together, these findings suggested that LINC01094 could be a therapeutic target and that using anti‐LINC01094 treatment (e.g., TEPP‐46) could be a promising strategy to suppress BC progression.

## 3 Discussion

As one of the key characteristics of malignant tumors, metabolic reprogramming alters the glucose metabolic pattern to favor a more aggressive phenotype and a stronger resistance to therapies, thereby hindering the development of effective therapeutic strategies for BC.^[^ ^33^ ^]^ Studies have implicated that lncRNAs play crucial roles in various types of cancer.^[^ ^34^ ^]^ However, the precise mechanism by which lncRNAs regulate glucose metabolism in cancer (including BC) merits further investigation. Herein, we identified an lncRNA, LINC01094, to be associated with energy metabolism. We elucidated a novel mechanism whereby LINC01094 could promote glycolysis and the progression of BC by acting as a molecular scaffold to recruit the PKM2/JMJD5 complex. These results highlighted LINC01094 as a potential therapeutic biomarker for BC.

We selected LINC01094, which was closely associated with an adverse prognosis in patients suffering from BC, for further investigation. LINC01094 expression was found to be markedly upregulated in BC tissues from patients with LNM, and could be a biomarker for predicting LNM in patients suffering from BC. Next, we explored the upstream regulatory mechanism of LINC01094 overexpression. Growing evidence indicates that epigenetic modifications play important roles in tumorigenesis and cancer progression.^[^ ^35^ ^]^ As the most abundant post‐transcriptional epigenetic modification of RNAs in eukaryotes, m^6^A has been demonstrated to exert critical molecular functions in modulating lncRNAs.^[^ ^36^, ^37^ ^]^ m^6^A is installed by methyltransferases (known as “writers”), which catalyze m^6^A modification via methyltransferase domains.^[^ ^38^ ^]^ We identified that the writer METTL14 promoted the m^6^A modification in LINC01094. m^6^A‐methylated RNAs recruit m^6^A readers, which have conserved m^6^A‐binding domains that can recognize m^6^A modifications specifically, to exert their biological functions.^[^ ^39^ ^]^ Therefore, m^6^A readers represent intermediaries for m^6^A modification of RNAs and determine their fates. As a post‐transcriptional enhancer, IGF2BP2 recruits RNA stabilizers to increase RNA stabilization in an m^6^A‐dependent manner.^[^ ^40^ ^]^ We reported that METTL14‐mediated m^6^A modification significantly upregulated LINC01094 expression in BC via IGF2BP2‐dependent stabilization.

Only a small fraction of lncRNAs have been well‐characterized in terms of function. However, they have been demonstrated to be dysregulated extensively in human malignancies, and they control essential metabolic regulatory networks by directly binding to one or more protein partners.^[^ ^41^ ^]^ To explore the molecular regulatory mechanism of LINC01094 in BC, we employed comprehensive identification of RBPs by RNA pull‐down assay to authenticate the composition and dynamics of ad hoc binding of RBP complexes with LINC01094. AGO2 protein was not enriched in the pull‐down product captured by LINC01094, so we ruled out the possibility that LINC01094 functioned as a ceRNA by “sponging” miRNAs. Subsequently, we identified that the 1–400‐nt and 1421–2186‐nt segments of LINC01094 could bind to PKM2, and these two sites that could bind to PKM2 monomers were close to each other. Furthermore, we demonstrated that LINC01094 increased the dimeric form of PKM2. Taken together, these findings suggested that LINC01094 could bind to PKM2 monomers physically and promote the formation of PKM2 dimers.

As the final rate‐limiting enzyme in glycolysis, PKM2 exists in inactive monomers, less active dimers, or active tetramers.^[^ ^42^ ^]^ The tetrameric PKM2 redirects glucose‐derived carbons toward adenosine‐triphosphate generation via OXPHOS. The dimeric PKM2 converts glucose metabolism to macromolecular anabolism via aerobic glycolysis. However, it can also enter the nucleus and interact with various transcription factors to support the Warburg effect and tumor progression by acting as the essential co‐activator to modulate transcription factors in the nucleus.^[^ ^43^ ^]^ Studies have shown that the dioxygenase JMJD5 can bind directly to PKM2 and promote the translocation of PKM2 into the nucleus by increasing its dimerization.^[^ ^28^ ^]^ Our data demonstrated that LINC01094 promoted the formation of PKM2/JMJD5 complexes by acting as a molecular scaffold, thereby promoting the conformational transformation of dimeric PKM2 and increasing its nuclear translocation.

It has been reported that PKM2 in the nucleus can bind to HIF‐1α, β‐catenin, or other transcription factors to regulate their transcriptional activities.^[^ ^44^ ^]^ As a key factor responding to hypoxic stress, HIF‐1α has been implicated in regulating glucose homeostasis by transcriptionally activating the genes involved in energy metabolism, thereby yielding increased glycolysis and promoting tumor progression.^[^ ^45^ ^]^ β‐catenin is a subunit of the cadherin protein complex. β‐catenin participates in regulating the coordination of cell‐cell adhesion and acts as an intracellular signal transducer in the Wnt signaling pathway.^[^ ^46^ ^]^ Consistently, we revealed that LINC01094 enhanced the binding of PKM2 to HIF‐1α and β‐catenin, and significantly promoted the expression of HIF‐1α target genes (PKM2, LDHA, GLUT1, and PDK1) as well as genes regulated by β‐catenin (CCND1 and c‐Myc). We concluded that LINC01094 increased the nuclear distribution of PKM2, thus triggering the transactivation of HIF‐1α and β‐catenin to their target genes.

A reprogrammed energy metabolic pattern generates sufficient materials for macromolecular biosynthesis and induces a more acidic environment for cancer cells to meet the needs for rapid proliferation and an invasive phenotype of tumor cells.^[^ ^47^ ^]^ Our data indicated that LINC01094 promoted glycolytic metabolism by regulating PKM2 and enhanced the aggressive growth and metastasis of BC cells markedly in vitro and in vivo. Next, we further evaluated the possible therapeutic potential of LINC01094 by intraperitoneal injection of TEPP‐46 into mice. TEPP‐46 is a small‐molecule activator that inhibits the formation of dimeric PKM2. TEPP‐46 treatment inhibited the interaction between PKM2 and its regulated transcriptional activators significantly.^[^ ^48^ ^]^ Therefore, the downstream pathway regulated by LINC01094 can be blocked using TEPP‐46. In vitro and in vivo assays indicated that TEPP‐46 repressed the promoted aerobic glycolysis and progression of BC induced by LINC01094. These findings elucidated the mechanism by which LINC01094 regulates the metabolic reprogramming of BC, providing new solutions for BC treatment.

Our study provides critical insights into the role of LINC01094 in the energy metabolism and tumor progression of BC. However, two key limitations warrant acknowledgment. First, our mechanistic validation was conducted primarily in TNBC models, a strategic choice driven by the urgent need to address this aggressive subtype. However, the generalizability of the functional mechanisms of LINC01094 across other molecular subtypes is unvalidated. Future studies should evaluate its subtype‐specific regulatory networks systematically to clarify its relevance across all subtypes of BC. Second, our reanalysis of TCGA‐BRCA data (*n* = 1073) supports the association between increased LINC01094 expression and worse overall survival. However, practical constraints (tissue‐degradation risks in archival samples and insufficient follow‐up time to capture mortality events in our cohort) prevented robust survival analyses using fresh specimens. This limitation reflects a broader challenge in lncRNA research, whereby longitudinal studies with rigorously preserved tissues are essential, though logistically demanding. We have initiated long‐term collaborations to address this knowledge gap and aim to prioritize survival validation in future prospective cohorts. These focused refinements will strengthen the translational relevance of our findings while advancing subtype‐specific therapeutic strategies.

Looking ahead, a major focus of our future research will be to translate these findings into clinical applications. This approach will involve the development of LINC01094‐specific therapeutics, such as small‐molecule inhibitors targeting the LINC01094‐PKM2 interactions identified in our study. Moreover, we plan to explore the potential of combining LINC01094 inhibition with existing treatment modalities to achieve synergistic anti‐tumor effects. Preclinical testing in patient‐derived models, followed by early‐phase clinical trials, will be essential to evaluate the efficacy, specificity, and safety of these interventions. Ultimately, this approach will not only help assess the broader applicability of LINC01094, but also identify potential biomarkers for predicting patient responses to LINC01094‐targeted therapies.

**Figure 8:**
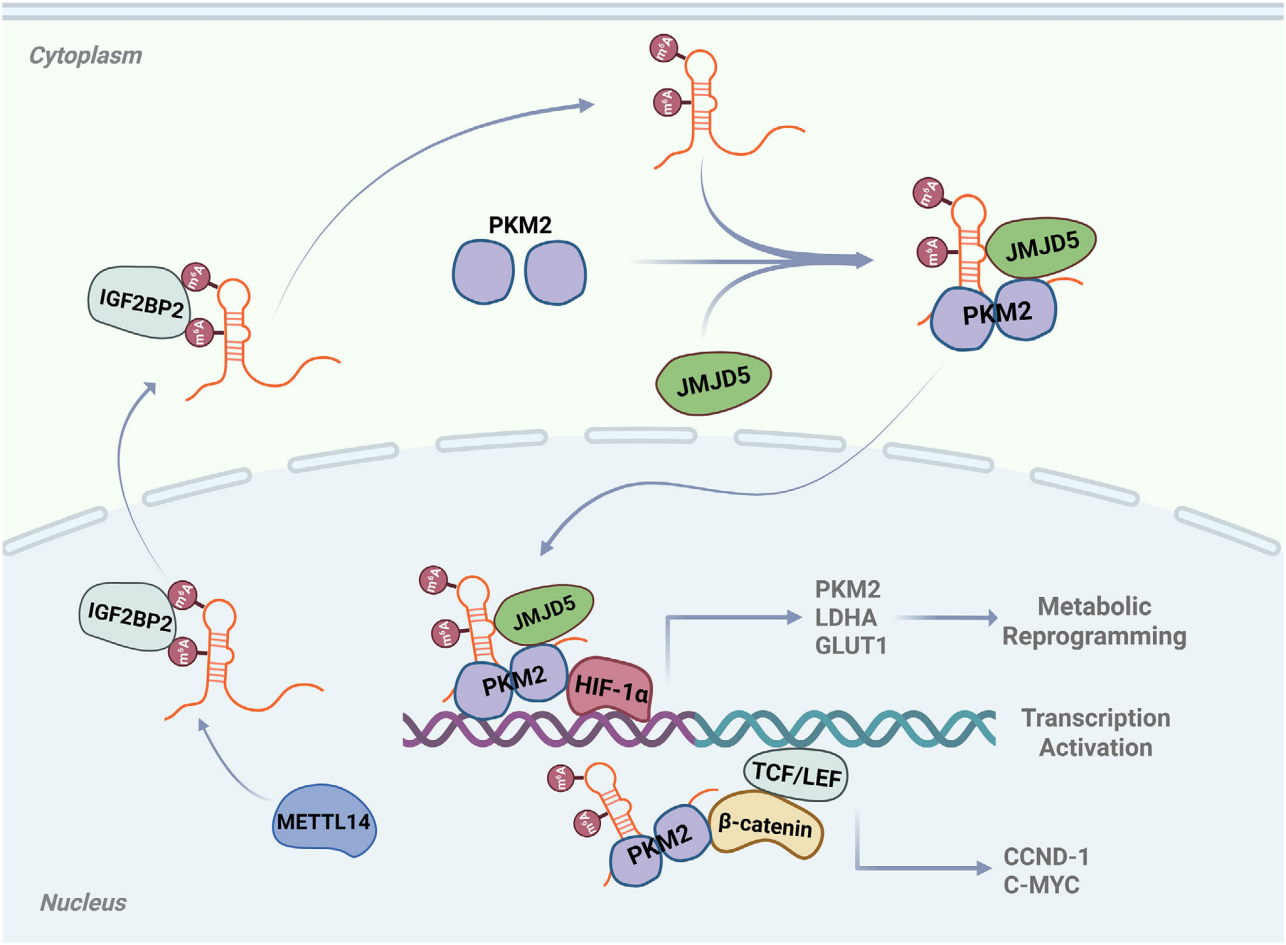
N^6^‐Methyladenosine‐modified LINC01094 induces glucose metabolic reprogramming in BC by recruiting the PKM2/JMJD5 complex. METTL14/IGF2BP2‐mediated N^6^‐methyladenosine (m^6^A) modification promotes the upregulation of LINC01094 in BC. LINC01094 interacts with the PKM2 monomer, facilitating PKM2 dimer formation. Additionally, LINC01094 serves as a flexible scaffold mediating the assembly of the PKM2/JMJD5 complex, further stabilizing the PKM2 dimer and enhancing its nuclear translocation. This process facilitates the PKM2‐induced transcriptional activity of HIF‐1α/β‐catenin, ultimately triggering a cascade in aerobic glycolytic and cancer progression.

In conclusion, our investigation uncovered a novel functional link between m^6^A‐modified lncRNAs and PKM2 conformational transition in the regulation of aerobic glycolysis and progression of BC. LINC01094 is expected to become a new prognostic marker and attractive therapeutic target for BC, and may lay a theoretical foundation for achieving more accurate and “personalized” treatments for patients suffering from BC (**Figure** **8**).

## 4 Experimental Section

### Human Tissue Specimens and Cell Lines

A total of 90 fresh BC tissues were collected from BC patients across two independent cohorts (Cohort 1: *n* = 45; Cohort 2: *n* = 45) at the Department of Pathology, Qilu Hospital, Shandong University (Jinan, China). The detailed patient information is presented in Table S1 (Supporting Information). All specimens were snap frozen and stored in liquid nitrogen. The research was approved by the Ethical Committee of the School of Basic Medical Sciences, Shandong University (Approval No. ECSBMSSDU 2018‐1‐006, ECSBMSSDU2023‐1‐52), and was conducted in accordance with the ethical guidelines of the World Medical Association Declaration of Helsinki.

BC cell lines, including MDA‐MB‐231, MDA‐MB‐468, MCF‐7, and BT‐474 cells, were obtained from the ATCC. All the cells were cultured in medium supplemented with 10% fetal bovine serum (FBS; BI, USA) at 37 °C in a humidified atmosphere with 5% CO_2_.

### Cell Transfection and Lentiviral Transduction

Cells were transfected with INTERFERin Reagent (Polyplus‐transfection, Inc., New York, USA) for siRNAs and ASOs (RiboBio Guangzhou, China) or with TurboFect Reagent (Thermo Fisher Scientific, Waltham, MA, USA) for plasmids. BC cells were infected with the lentivirus vectors carrying LINC01094 shRNA (GenePharma, Shanghai, China). Forty‐eight hours after infection, puromycin (1 µg mL^−1^; Solarbio, Beijing, China) was added to the culture medium and incubated with the cells for at least two to three passages to establish stable knockdown cell lines.

### RNA Extraction and Real‐Time (Quantitative) Reverse Transcription PCR (qRT‐PCR)

Total RNA was extracted from BC tissue and cell lines by the TRIzol Reagent (Thermo Fisher Scientific) and quantified by NanoDrop One/One (Thermo Fisher Scientific). One microgram of RNAs was reverse transcribed into cDNA using a ReverTra Ace qPCR RT Kit (TOYOBO, Osaka, Japan). Real‐time (Quantitative) PCR (qPCR) was performed with the SYBR Green Realtime PCR Master Mix (Roche, Basel, Switzerland) on a Bio‐Rad Real‐Time PCR system (Bio‐Rad, USA) following the manufacturer's instructions. Primers used for qPCR are listed in Table S2 (Supporting Information).

### Subcellular Fractionation

Nuclear and cytoplasmic RNA were isolated respectively by NE‐PER Nuclear and Cytoplasmic Extraction Reagents (Thermo Fisher Scientific, Waltham, MA, USA) according to the manufacturer's protocol. Then, qRT‐PCR was conducted to analyze the expression of LINC01094. GAPDH and U6 acted as internal references for cytoplasmic and nucleus.

### Fluorescence In Situ Hybridization (FISH) and Immunofluorescence Analysis

The Cy3‐labeled probe for LINC01094 was designed and synthesized by RiboBio (Guangzhou, China). RNA‐FISH was performed with the Ribo FISH Kit (RiboBio, Guangzhou, China) in accordance with the manufacturer's protocol. In brief, BC cells cultured on chamber slides were fixed with 4% paraformaldehyde (PFA) for 10 min, followed by three PBS washes. The fixed cells were further permeabilized with 0.5% Triton X‐100 in PBS at 4 °C for 5 min and then washed with PBS. Subsequently, the cells were incubated overnight at 37 °C with 0.5 µM FISH probes (RiboBio) in hybridization buffer. The U6 and 18s probe (RiboBio, Guangzhou, China) were used as internal controls. Following hybridization, slides were washed five times with hybridization wash buffer at 42 °C. Finally, nuclei were counterstained with DAPI, and images were captured using a confocal laser scanning microscope (LSM980, Zeiss).

For IF analysis, cells subjected to specific treatments were fixed with 4% PFA at RT for 10 min, then permeabilized with 0.5% Triton X‐100 in PBS for 5 min. Subsequently, cells were blocked with goat serum at RT for 30 min to minimize nonspecific binding and incubated overnight with primary antibodies at 4 °C, followed by incubation with species‐specific secondary antibodies (anti‐rabbit/mouse IgG (H+L), cross‐adsorbed, conjugated with Alexa Fluor 488 or 594) at 37 °C for 1 h. Nuclei were counterstained with DAPI, and cells were washed three times with PBS. Finally, use a confocal laser scanning microscope (LSM980, Zeiss) for IF image acquisition.

### Cell Proliferation Assay

Cell proliferation was measured with CCK‐8 and EdU assays. The CCK‐8 assay was performed using the Cell Counting Kit‐8 (CCK‐8; Topscience, Shanghai, China), and the EdU assay was performed following the protocols of YF488/555/594/647A Click‐iT EdU Imaging Kits (US Everbright Inc., Suzhou, China).

### Transwell Assay

The transwell migration/invasion assay was performed using 24‐well plates containing 8.0 µm polycarbonate membrane (Corning, Life Science, NY, USA) according to the manufacturer's instructions. Briefly, chambers with Matrigel (Corning, Life Science, NY, USA) (for invasion assay) or without Matrigel (for migration assay) were placed into 24‐well plates, which were covered by the DMEM medium containing 10% FBS. BC cells suspended in serum‐free medium were added to the upper chamber. After 37 °C for 16 h for migration assay or 24 h for invasion assay, cells that traversed to the bottom of the chamber were fixed with 4% PFA, and stained with crystal violet (Sigma‐Aldrich, V5265). Images were captured using the Nikon microscope, and cell counts were quantified with ImageJ.

### Detection of Glucose Uptake and Lactate Levels

Glucose uptake and lactate levels were detected, respectively using Glucose Uptake Colorimetric Assay Kit (catalog nos. ab136955, Abcam) and Lactic Acid Assay Kit (Abbkine, #ktb1100) according to the manufacturer's instructions. BC cells were seeded in 6‐well plates and incubated at 37 °C for 24 h. After transfection or other treatments, cells were transferred to 96‐well plates at a density of 3000 cells per well and serum‐starved for 12 h following cell attachment. Subsequently, the cells were washed three times with PBS and incubated in KRPH/2% BSA for 40 min. Then the cells were treated with 2‐DG for 20 min. Finally, glucose uptake was assessed by kinetic measurement of optical density (OD) at 412 nm, in accordance with the manufacturer's protocol.

For measurement of lactate production, cell supernatant (10 µL) was mixed with 40 µL Lactate Assay Buffer and 50 µL Working Reagent in a 96‐well plate, then incubated at 37 °C for 30 min protected from light. Absorbance was measured at 450 nm using a Multiskan SkyHigh Microplate Reader, with results normalized to cell number.

### Oxygen Consumption Rate (OCR) and Extracellular Acidification Rate (ECAR)

OCR and ECAR were measured using a Seahorse XFe96 Analyzer from Seahorse Bioscience (MA, USA). Experimental protocols adhered to the instructions provided with the Seahorse XF Cell Mito Stress Test kit (OCR) and the Seahorse XF Glycolysis Stress Test kit (ECAR), both from Agilent (Santa Clara, CA, USA). Briefly, BC cell lines were seeded in a Seahorse XF96 Cell Culture Microplate (Agilent) at a density of 1  ×  10^4^ cells per well and incubated at 37 °C overnight to allow attachment. Subsequently, cells were equilibrated with the detection medium in a non‐CO₂ incubator before analysis. During OCR measurements, oligomycin A (1.5 µM), FCCP (1 µM), and rotenone/antimycin A (0.5 µM) were sequentially injected. For ECAR assessment, glucose (10 mM), oligomycin A (1 µM), and 2‐DG (50 mM) were administered sequentially. OCR and ECAR were calculated according to the manufacturer's instructions using Seahorse Wave software.

### Actinomycin D (ActD) Assay

For mRNA stability assays, MDA‐MB‐231 and MDA‐MB‐468 cells were transiently transfected with specified siRNAs or plasmids using Lipofectamine 2000. Following a 48‐h incubation period, cells were treated with ActD (5 µg mL^−1^; Sigma‐Aldrich), and total RNA was harvested at 0‐, 4‐, and 8‐h post‐treatment. The samples were then subjected to RNA extraction and RT‐qPCR analysis as described above, with results normalized to GAPDH expression levels.

### Nuclear and Cytoplasmic Protein Fractionation and Western Blot

Total protein was extracted using the pre‐mixed RIPA Lysis Buffer (Beyotime, Shanghai, China) with the protease inhibitor (NCM Biotech, Suzhou, China). Nuclear and cytoplasmic fractions were isolated by the Nuclear and Cytoplasmic Protein Extraction Kit (Beyotime, Shanghai, China) following the manufacturer's protocol. Protein concentration was quantified using the NCM BCA Protein Assay Kit (NCM Biotech, Suzhou, China). GAPDH and Lamin B1 were used as protein loading controls for cytoplasmic and nuclear fractions, respectively. The information on antibodies is listed in Table S2 (Supporting Information).

### RNA Pull‐Down Assay

The biotin‐labeled LINC01094 and antisense strands RNA were synthesized in vitro using Bio‐16‐UTP (Thermo Fisher Scientific, IL, USA) according to the protocols of the MEGAscript T7 High Yield Transcription Kit (Thermo Fisher Scientific, IL, USA), followed by purification using the MEGAclear Transcription Clean‐Up Kit (Thermo Fisher Scientific, IL, USA). For RNA‐protein interaction analysis, 3 µg of biotinylated RNA was incubated with 1 mg of protein lysate extracted from BC cells for 3 h and then mixed with pre‐washed M‐280 Streptavidin Dynabeads (Invitrogen) for 2 h. Finally, proteins specifically pulled down by biotin RNA were eluted from the beads and identified by western blot or silver staining followed by mass spectrometry.

### RNA Immunoprecipitation (RIP) Assay

The RIP assay was conducted following the protocols of the MagnaRIP RNA Binding Protein Immuno‐precipitation Kit (Millipore, MA, USA). Briefly, magnetic beads were incubated with 5 µg anti‐PKM2, anti‐JMJD5 or anti‐IgG antibodies for 30 min, followed by immunoprecipitation with BC cell lysates overnight at 4 °C. Finally, the co‐precipitated RNAs were purified, and the amount of LINC01094 in the eluate was analyzed by RT‐qPCR.

### Co‐Immunoprecipitation (Co‐IP)

Cellular proteins from BC cells were collected and lysed with IP lysis buffer (Thermo Fisher Scientific, IL, USA) [25 mM Tris‐HCl pH 7.4, 150 mM NaCl, 1% NP‐40, 1 mM EDTA, 5% glycerol] supplemented with 1× protease inhibitor cocktail (NCM Biotech, Suzhou, China). Lysates were then incubated with 2 µg primary antibodies or IgG and 20 µL Protein A/G PLUS‐Agarose (Santa Cruz Biotechnology, USA) at 4 °C overnight. Beads were washed five times using the lysis buffer to collect the immunoprecipitates. The samples were then subjected to SDS‐polyacrylamide gel electrophoresis and analyzed by immunoblotting with the specified antibodies.

### Methylated RNA Immuneprecipitation (MeRIP) Assay

The MeRIP assay was performed with the Magna m^6^A MeRIP Kit (Merck Millipore) to quantify the m^6^A modification level of LINC01094. Total RNA was extracted from cultured BC cells using the TRIzol Reagent (Thermo Fisher Scientific, IL, USA). Subsequently, the RNA was fragmented and immunoprecipitated with Protein A/G magnetic beads conjugated with 10 µg anti‐m^6^A or anti‐IgG antibodies at 4 °C for 2 h. Finally, RNA was eluted from the immunoprecipitates using a prepared elution buffer and purified with the RNA Purification Kit for subsequent qRT‐PCR analysis.

### DSS Crosslinking Assay

Disuccinimidyl suberate (DSS) crosslinker (Thermo Fisher Scientific, IL, USA) was used for protein crosslinking according to the manufacturer's instructions. Briefly, cells were incubated with 1 mM DSS at room temperature for 30 min, followed by the addition of 10 mM Tris to quench the reaction for 15 min. Subsequently, the crosslinked proteins were analyzed by western blotting.

### Dual Luciferase Reporter Assay

293T cells were seeded in 24‐well plates at 40% confluence for 12 h. Subsequently, cells were transiently transfected with LINC01094/pcDNA3.1 plasmid or ASOs using Lipofectamine 2000 transfection reagent (Invitrogen, USA) for 24 h. Following this, each well was co‐transfected with 1 µg HIF‐1α reporter plasmid or TOP/FOPflash reporter plasmid, along with 0.1 µg Renilla luciferase plasmid as an internal control. At 24 h post‐transfection, luciferase activity was measured according to the manufacturer's protocol of the Dual‐Luciferase Reporter Assay Kit (Vazyme, Nanjing, China). The relative luciferase activity was determined by normalizing firefly luciferase (LUC) signals to Renilla luciferase (REN) signals.

### Animal Models

BALB/c athymic nude mice (female, 3 weeks old) were obtained from Weitong Lihua Biotechnology (Beijing, China). Subsequently, xenograft tumor and hematogenous metastasis models were established by either subcutaneous implantation of MDA‐MB‐231 cells into the mammary fat pads or intravenous injection via the tail vein, respectively. The calculation of tumor volumes was conducted using the formula V = (Tumor length × Width^2^)/2. At the end of a 5‐week (xenograft tumor model) or 8‐week (hematogenous metastasis model) experimental period, the mice were euthanized, and their tumors, livers, and lungs were harvested. Half of each tumor, along with the entire lungs and livers, were subjected to histological evaluation through hematoxylin and eosin (HE) staining. The remaining tumor samples were promptly frozen and stored for subsequent RNA and protein extraction. For therapeutic assay, 3×10^6^ MDA‐MB‐231 cells were subcutaneously implanted into the mammary fat pads of mice. Two weeks later, mice with the same‐sized tumor were randomly divided into two treatment groups: the vehicle group and the TEPP‐46 (40 mg kg^−1^) group. TEPP‐46 was purchased from MedChemExpress LLC (MCE, New Jersey, USA) and administered daily by intraperitoneal injection for three weeks. Tumor volumes were recorded by caliper measurements at 5‐day intervals. At the end of treatment, the mice were euthanized, and the therapeutic effect of TEPP‐46 was evaluated. The animal experiments were conducted in a blinded fashion and were approved by the Ethical Committee of the School of Basic Medical Sciences at Shandong University (Approval No. ECSBMSSDU2023‐2‐102).

### Statistical Analysis

All statistical analyses were performed using GraphPad Prism 9.0 (GraphPad Software, USA). Differences between the two groups were assessed by unpaired Student's t‐test. Comparisons among multiple groups were conducted through analysis of variance (ANOVA). The correlation between LINC01094 expression and clinicopathological parameters of BC patients was analyzed by the Pearson's chi‐square test. Survival curves were plotted by the Kaplan–Meier method and assessed using the log‐rank test. Data are presented as mean ± standard deviation (SD) of at least three independent experiments. A two‐tailed *p*‐value <0.05 was considered statistically significant (*p < 0.05, **p < 0.01, and ***p < 0.001).

### Ethical Approval

The research protocols were reviewed and approved by the Ethical Committee of the School of Basic Medical Sciences, Shandong University (Approval No. ECSBMSSDU 2018‐1‐006, ECSBMSSDU2023‐1‐52 for human subjects research and ECSBMSSDU2023‐2‐102 for animal experimentation).

## Conflict of Interest

The authors declare no conflict of interest.

## Author Contributions

G.P. led the project design and manuscript preparation. Z.W.J. was involved in study design and manuscript writing. W.M.Q. and G.Z.X. are co‐first authors. W.M.Q. contributed to study design, conducted experiments, analyzed data, and prepared figures. G.Z.X. conducted experiments and contributed to data interpretation. Z.R.N., Z.P., C.J.X., Z.H., and W.Y.W. were involved in conducting experiments. M.W. and Z.G. contributed equally to this work.

## Supporting information



Supporting Information

## Data Availability

The data that support the findings of this study are available from the corresponding author upon reasonable request.
