# Supplementary material for: METTL14‐Mediated M6A Modification of LINC01094 Induces Glucose Metabolic Reprogramming in Breast Cancer by Recruiting the PKM2/JMJD5 Complex
Source: Adv Sci (Weinh). 2025 Jun 5;12(35):e10386. doi: 10.1002/advs.202410386 (PMC12463113; doi:10.1002/advs.202410386)
Supplement: Supplementary file 1 — Supporting Information [file ADVS-12-e10386-s001.pdf]

ADVANCED SCIENCE
Open Access

Supporting Information

for *Adv. Sci.*, DOI 10.1002/advs.202410386

METTL14-Mediated M^{6}A Modification of LINC01094 Induces Glucose Metabolic Reprogramming in Breast Cancer by Recruiting the PKM2/JMJD5 Complex

Mengqi Wang, Zhaoxin Gao, Ruinan Zhao, Pan Zhou, Jingxi Chen, Hui Zhang, Yawen Wang, Wenjie Zhu and Peng Gao**

Supplementary figures

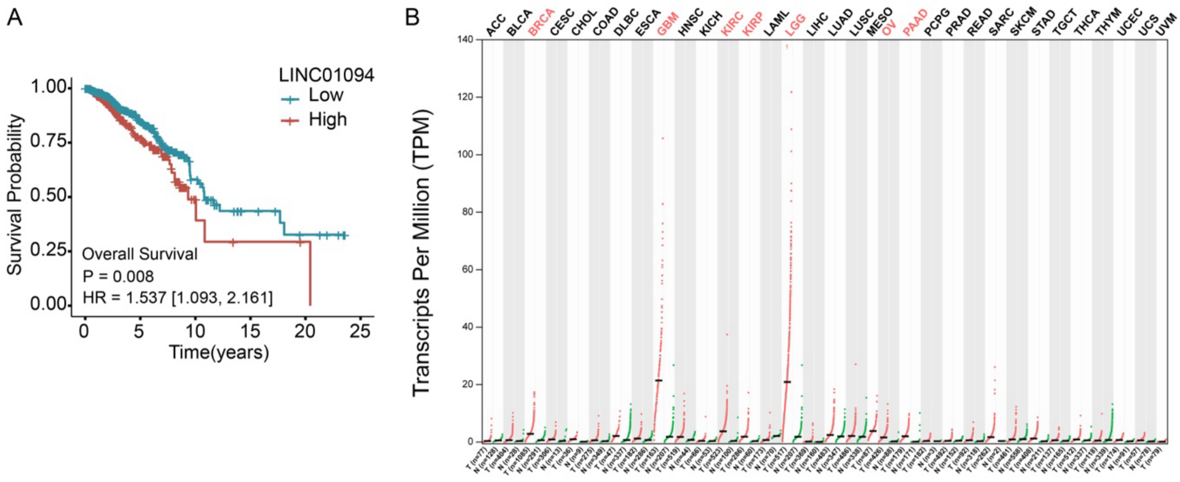

C

Database (Website)	Label	Coding probability
CPAT (http://lilab.research.bcm.edu/)	Noncoding	0.017480
CPC (http://cpc2.cbi.pku.edu.cn/)	Noncoding	0.117228

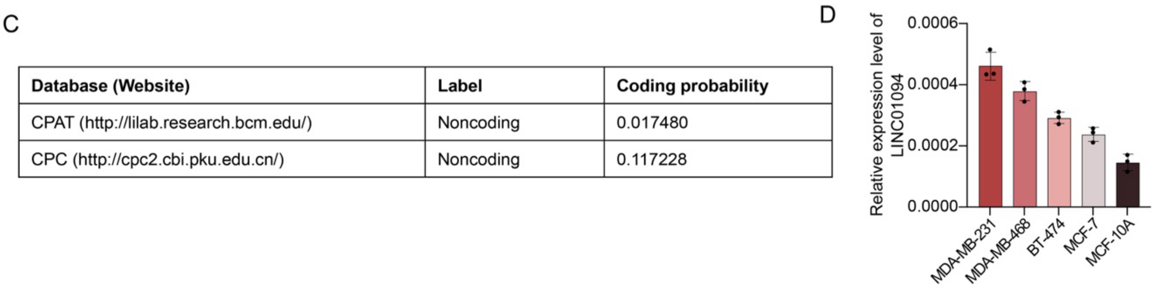

Figure S1. Expression and characterization of LINC01094.

(A) The association between LINC01094 expression and the survival of BC patients was analyzed using the TCGA dataset. Kaplan–Meier survival analysis using the log-rank test was employed to assess overall patient survival. **(B)** The analysis of TCGA cohort showed that LINC01094 was markedly up-regulated in various tumors, including breast cancer. **(C)** The bioinformatics tool CPAT and CPC was utilized to predict the coding potential of LINC01094. **(D)** qRT-PCR analysis of LINC01094 expression levels in MDA-MB-231, MDA-MB-468, MCF-7, BT-474, and MCF-10A. Results are presented as means ± SD by Student's t test.

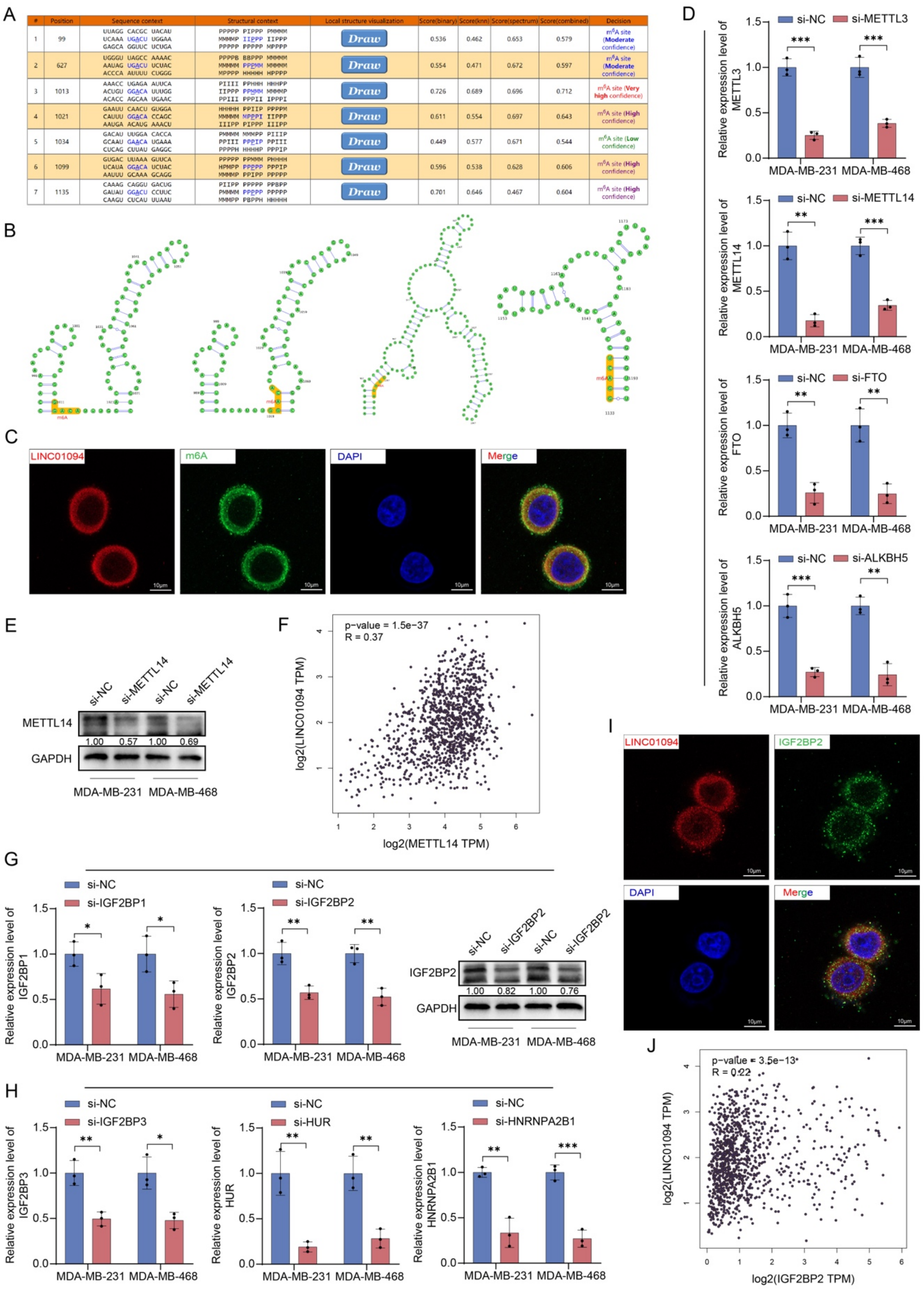

Figure S2. METTL14-mediated m⁶A modification stabilizes LINC01094 through IGF2BP2 in BC.

(A, B) The prediction of putative m^6A modification sites in LINC01094 sequences was carried out using SRAMP website. **(C)** We conducted RNA-FISH and immunofluorescence staining by using fluorescent probes targeting LINC01094 and antibody designed to specifically bind to m^6A-modified RNA molecules in MDA-MB-468 cells. The images were captured at an original magnification of ×630. Scale bars: 10 μm. **(D)** qRT-PCR assay was used to detect the knockdown efficiency of m^6A writers in BC cells. **(E)** Western blot assay was employed to assess the knockdown efficiency of METTL14 at the protein level in BC cells. **(F)** The database GEPIA was utilized to analyze the correlation between LINC01094 and METTL14. **(G, H)** qRT-PCR and western blot assays were used to assess the knockdown efficiency of m^6A readers in BC cells. **(I)** RNA-FISH and immunofluorescence staining was carried out using fluorescent probes targeting LINC01094 and an antibody specifically bind to IGF2BP2 in MDA-MB-468 cells. The images were captured at an original magnification of ×630. Scale bars: 10 μm. **(J)** The database GEPIA was utilized to analyze the correlation between LINC01094 and IGF2BP2. Results are presented as means ± SD; $*p < 0.05$; $**p < 0.01$; $***p < 0.001$ by Student's t test.

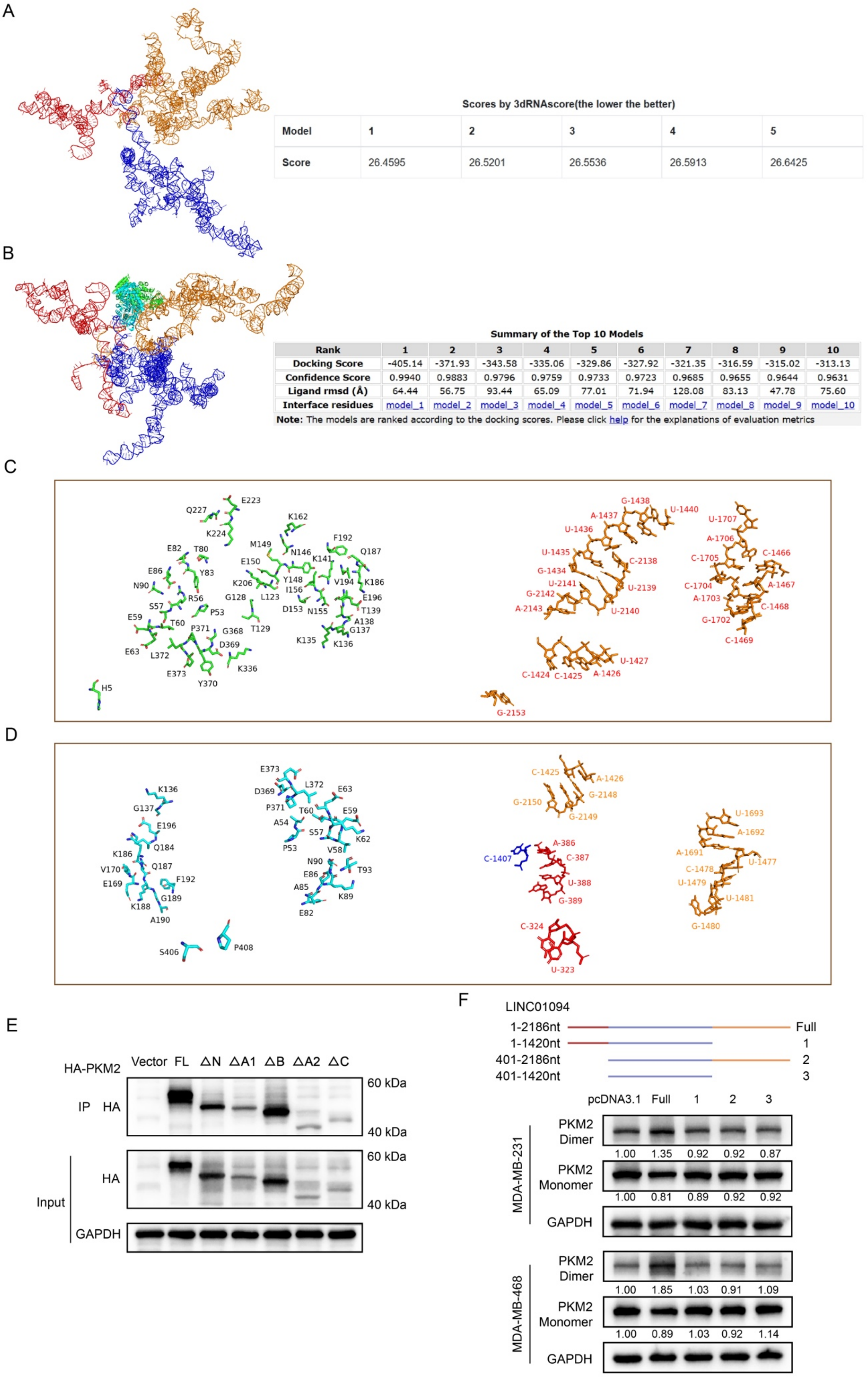

Scores by 3dRNAscore(the lower the better)

Model	1	2	3	4	5
Score	26.4595	26.5201	26.5536	26.5913	26.6425

Summary of the Top 10 Models

Rank	1	2	3	4	5	6	7	8	9	10
Docking Score	-405.14	-371.93	-343.58	-335.06	-329.86	-327.92	-321.35	-316.59	-315.02	-313.13
Confidence Score	0.9940	0.9883	0.9796	0.9759	0.9733	0.9723	0.9685	0.9655	0.9644	0.9631
Ligand rmsd (Å)	64.44	56.75	93.44	65.09	77.01	71.94	128.08	83.13	47.78	75.60
Interface residues	model_1	model_2	model_3	model_4	model_5	model_6	model_7	model_8	model_9	model_10

Note: The models are ranked according to the docking scores. Please click help for the explanations of evaluation metrics

Figure S3. LINC01094 engages with PKM2, promoting the formation of PKM2 dimers. **(A)** The 3D model of LINC01094 was constructed based on its sequence. The 3dRNAscore, where a lower score indicates a more reasonable structure, is presented in the table on the right-hand side. Model1's nucleic acid structure was chosen for subsequent molecular docking. The structure highlights 1-400nt in red, bases 1421-2186nt in orange, and bases 401-1420nt in blue, as indicated. **(B)** The Docking Score for the protein-nucleic acid interaction yielded an optimal result of -405.14, along with a Confidence Score of 0.9940. These values indicate a high level of reliability for the resulting complex model obtained from the docking process. The optimal model for the protein-nucleic acid interaction revealed the protein dimer binding between the red and orange nucleotide sequences. **(C)** The distribution of interface residues between the protein A chain of PKM2 dimer (the left panel) and LINC01094 nucleic acid (the right panel). **(D)** The distribution of interface residues between the protein B chain of PKM2 dimer (the left panel) and LINC01094 nucleic acid (the right panel). **(E)** Western blot analysis assessing the immunoprecipitation efficiency of HA-tagged full-length or truncated PKM2 in RIP assays. **(F)** DSS crosslinking–western blot analysis demonstrated that, in contrast to full-length LINC01094, the truncated variant lacking the PKM2-binding sequence failed to promote PKM2 dimer formation.

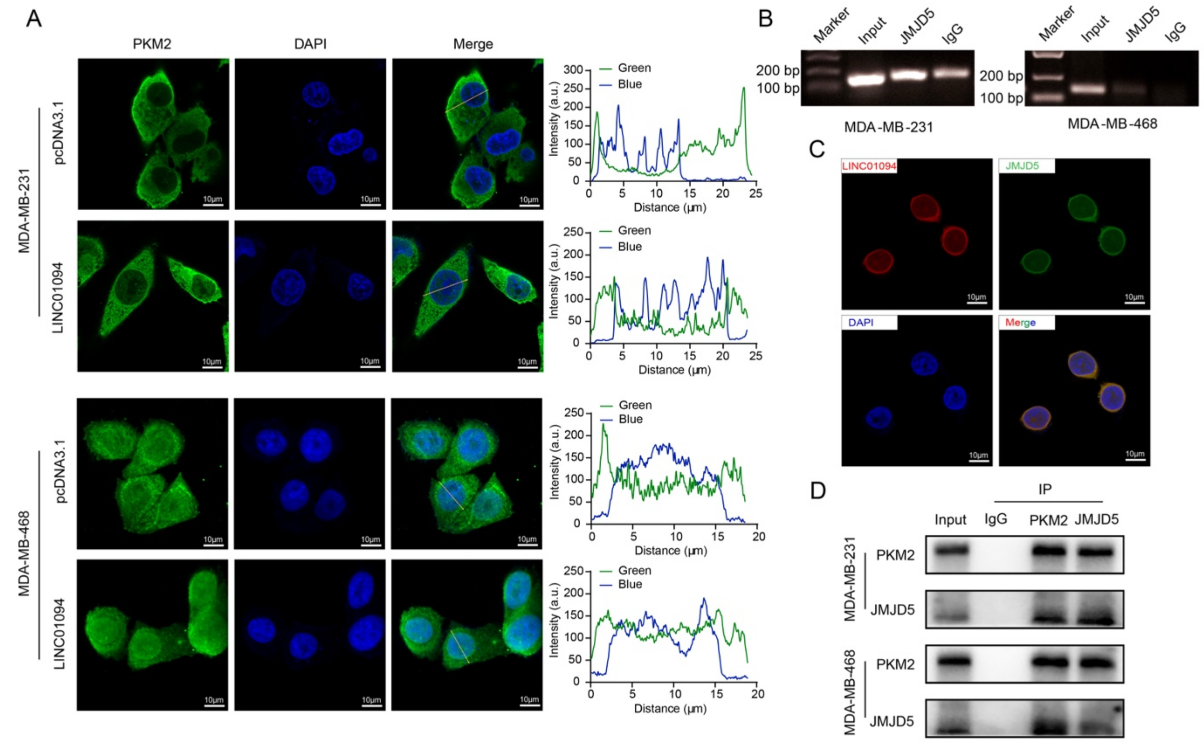

Figure S4. LINC01094 facilitates the formation of PKM2/JMJD5 complexes and enhances the translocation of PKM2 to the nucleus.

(A) Immunofluorescence staining verified that up-regulating the expression of LINC01094 markedly improved the nuclear localization of PKM2. The images were captured using an original magnification of ×630. Scale bars: 10 μm. **(B)** The detection of LINC01094 pulled down by JMJD5 protein in the RIP assay was performed using qRT-PCR, followed by DNA electrophoresis for verification. **(C)** RNA-FISH and immunofluorescence staining unequivocally confirmed the co-localization of LINC01094 and JMJD5 within MDA-MB-468 cells. The images were captured using an original magnification of ×630. Scale bars: 10 μm. **(D)** Co-IP assays confirmed the co-localization of PKM2 and JMJD5 in BC cells.

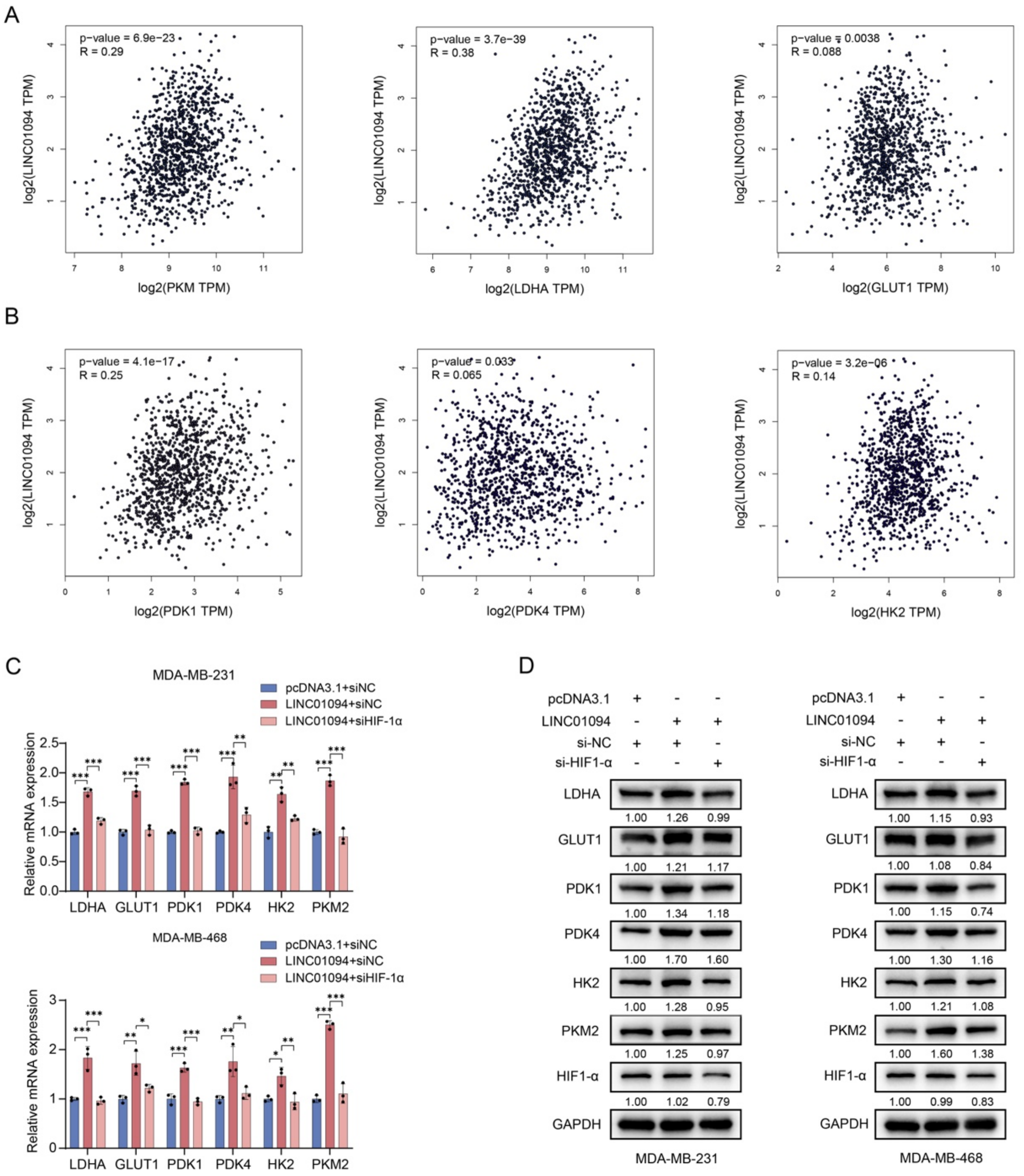

Figure S5. LINC01094 promotes the interaction of PKM2 with HIF1-α and β-catenin and facilitates their transactivation activity.

(A, B) Analysis of the GEPIA database revealed a positive correlation between LINC01094 and the HIF-1α targeted genes. **(C, D)** qRT-PCR **(C)** and western blot assays **(D)** confirmed that the promotion effect of LINC01094 on the downstream regulatory genes of HIF-1α could be reversed by knocking down HIF-1α. Data are presented as mean ± SD; statistical significance is indicated (*p < 0.05; **p < 0.01; ***p < 0.001) by ANOVA.

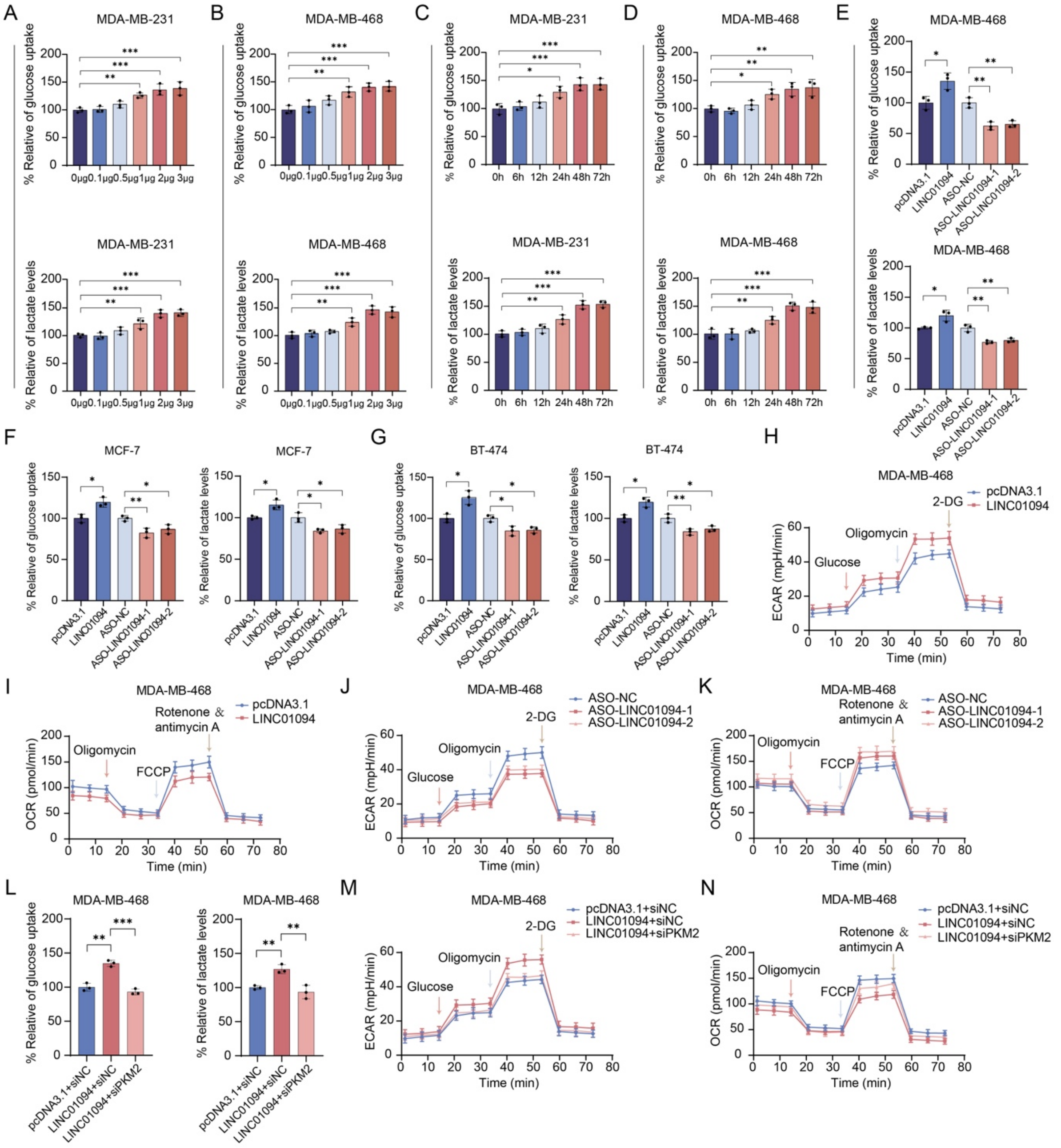

Figure S6. LINC01094 promotes aerobic glycolysis in BC.

(A, B) BC cells were transfected with LINC01094 plasmid at 0, 0.1, 0.5, 1, 2, or 3 μg, followed by assessment of glucose uptake and lactate production. The results showed that 2 μg induced the strongest response, with no additional enhancement observed at higher doses. **(C, D)** The impact of LINC01094 overexpression on glucose uptake and lactate production in BC cells were evaluated at 0, 6, 12, 24, 48, and 72 hours post-transfection. The effects of LINC01094 were initially observed at 24 hours and reached stabilization by 48 hours. **(E-N)** The levels of

glucose uptake, lactate production **(E-G and L)**, ECAR **(H, J, and M)**, and OCR **(I, K, and N)** were measured to detect the energy metabolism in BC cells. Data are presented as mean ± SD; statistical significance is indicated (*p < 0.05; **p < 0.01; ***p < 0.001) by Student's t test or ANOVA.

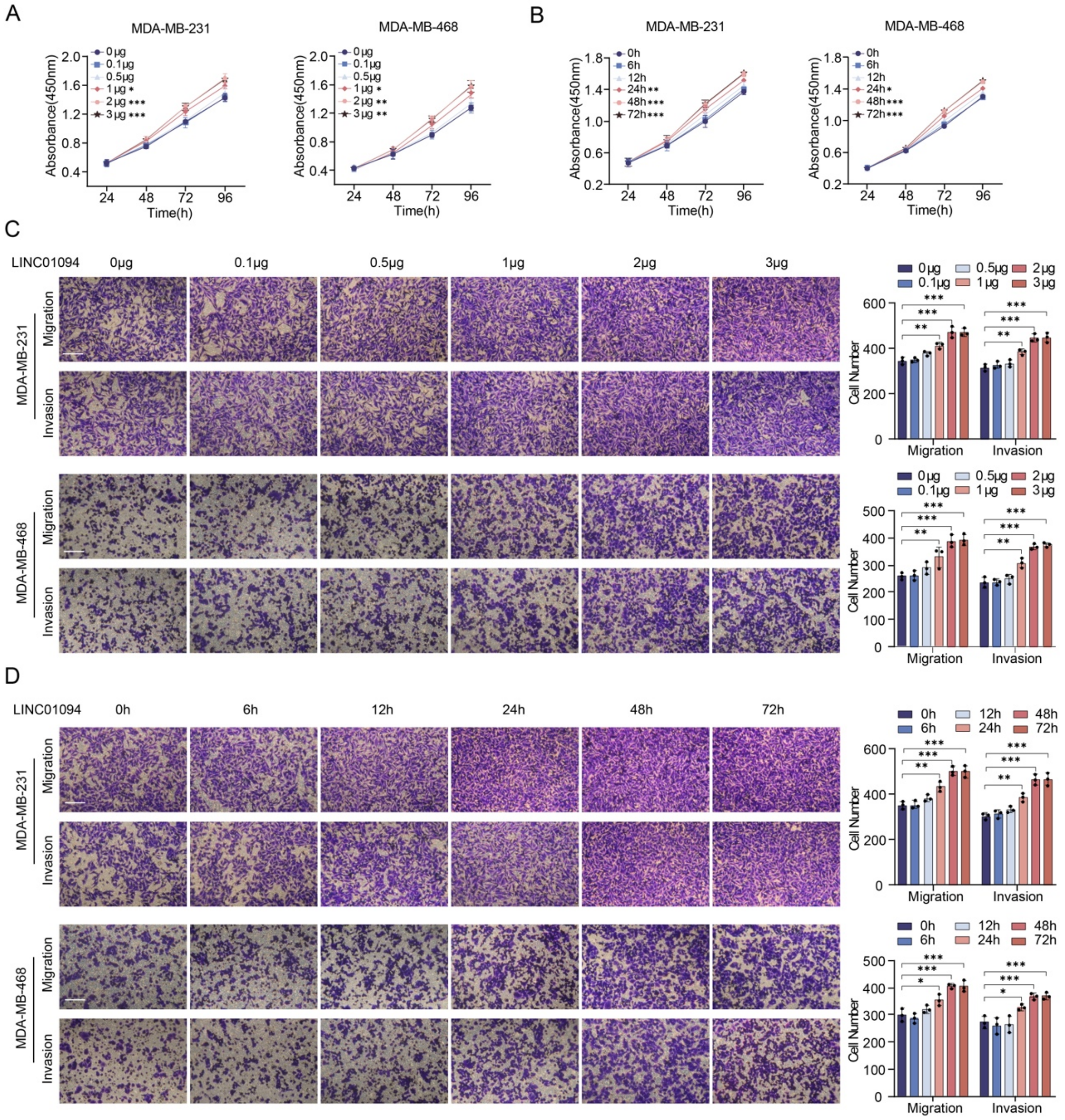

Figure S7. Dose-dependent and time-dependent effects of LINC01094 overexpression on tumor progression.

(A, B) The dose-dependent (0, 0.1, 0.5, 1, 2, 3 μg) and time-dependent (0, 6, 12, 24, 48, and 72 hours) effects of LINC01094 overexpression on BC cell proliferation were assessed by the CCK-8 assay. **(C, D)** The dose-dependent (0, 0.1, 0.5, 1, 2, 3 μg) and time-dependent (0, 6, 12, 24, 48, and 72 hours) effects of LINC01094 overexpression on BC cell migration and invasion were evaluated through transwell assays. Data are presented as mean ± SD; statistical significance is indicated (*p < 0.05; **p < 0.01; ***p < 0.001) by ANOVA.

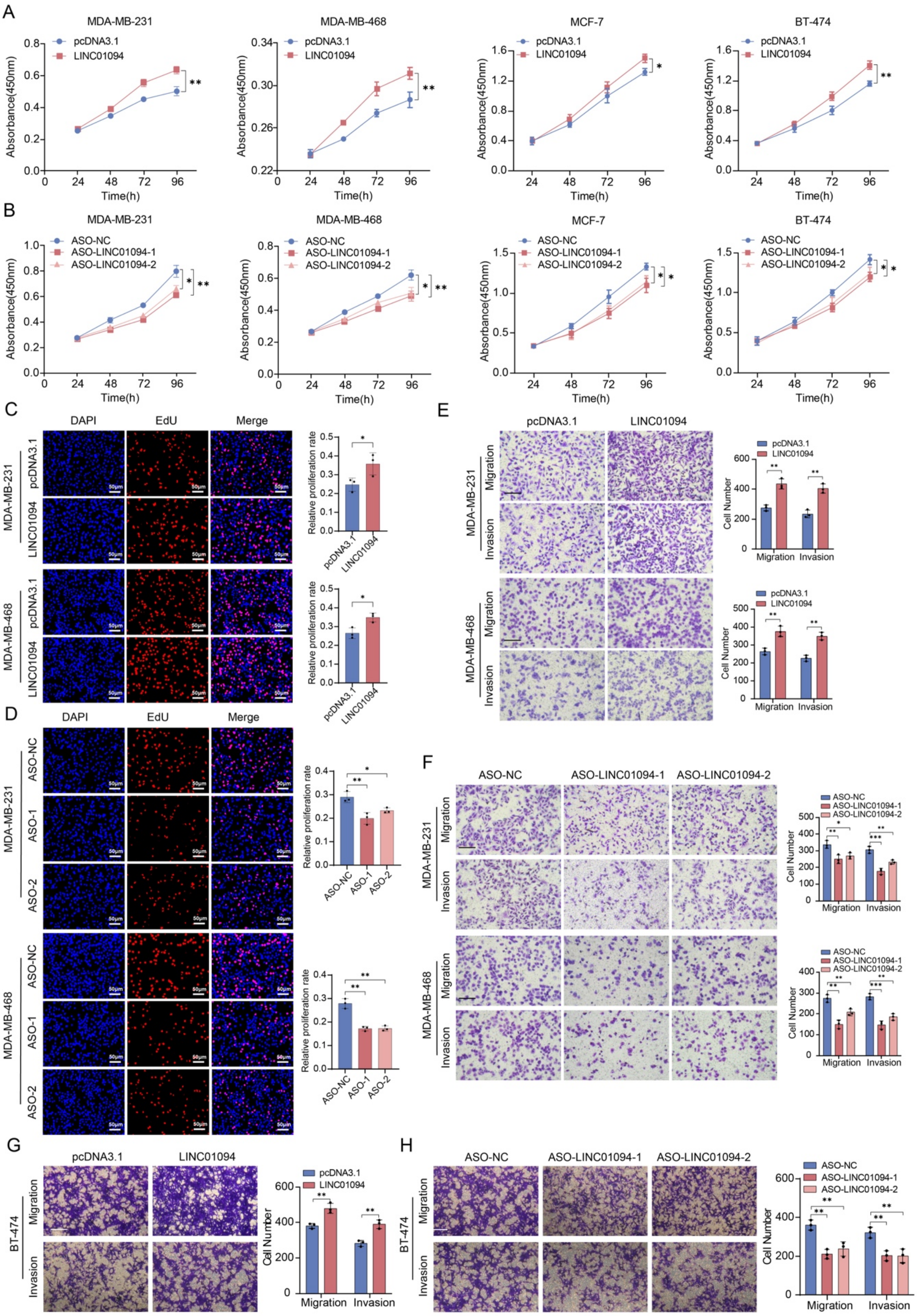

Figure S8. LINC01094 promotes tumor progression in BC.

(A-D) The CCK8 **(A, B)** and EdU **(C, D)** assays were employed to assess the influence of LINC01094 on the proliferative capacity of BC cells. Images were captured at 200× magnification. Scale bars: 50 μm. **(E-H)** Migration and invasion abilities of BC cells were assessed using transwell assays, and images were captured at 200× magnification. Scale bars: 100 μm. Data are presented as mean ± SD; statistical significance is indicated (*$p < 0.05$; **$p < 0.01$; ***$p < 0.001$) by Student's t test or ANOVA.

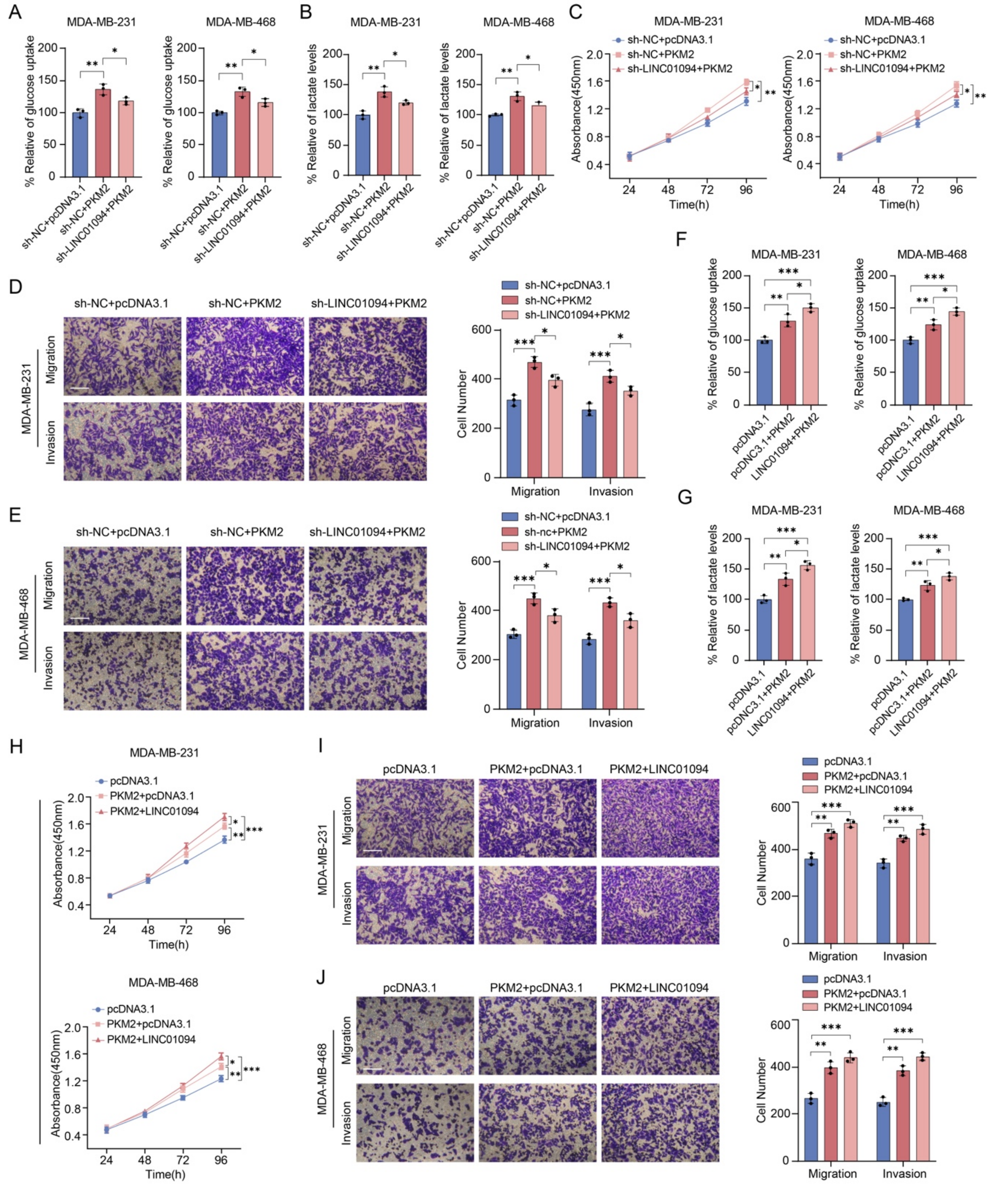

Figure S9. Role of LINC01094 in PKM2-mediated metabolic reprogramming and tumor progression.

(A, B) PKM2 overexpression enhances glucose uptake **(A)** and lactate production **(B)**, but these effects are reduced in LINC01094-knocked down cells. **(C-E)** PKM2 overexpression promotes cell proliferation **(C)**, migration, and invasion **(D, E)**, but these effects are weakened in

LINC01094-knockdown cells. **(F-J)** Co-overexpression of LINC01094 and PKM2 potentiates glucose metabolism and tumor progression compared to PKM2 overexpression alone, as shown by increased glucose uptake **(F)**, lactate production **(G)**, cell proliferation **(H)**, and migration/invasion **(I, J)**. Data are presented as mean ± SD; statistical significance is indicated (*$p < 0.05$; **$p < 0.01$; ***$p < 0.001$) by ANOVA.

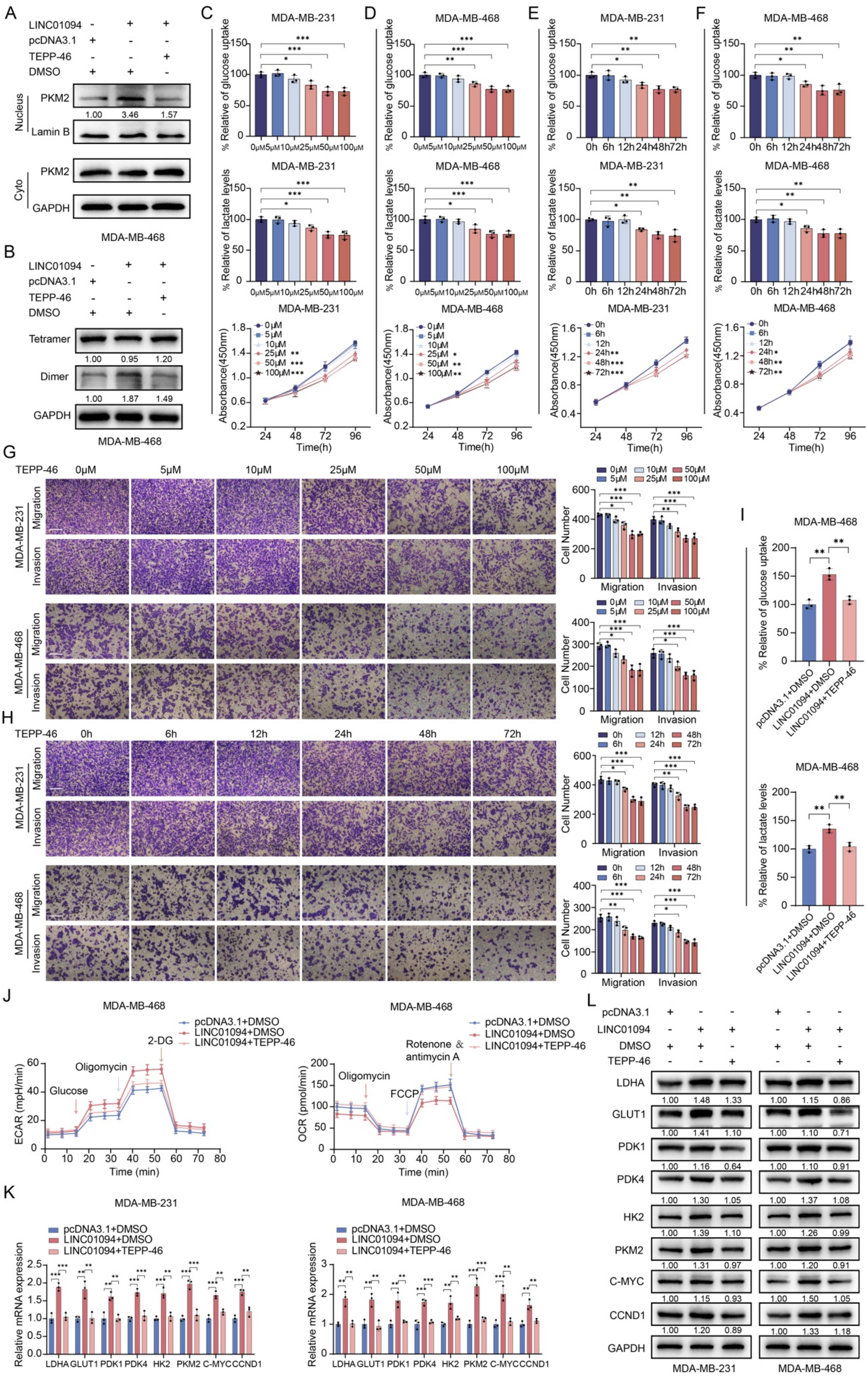
A
LINC01094 - + +
pcDNA3.1 + - -
TEPP-46 - - +
DMSO + + -
Nucleus
PKM2
1.00 3.46 1.57
Lamin B
Cyto
PKM2
GAPDH
MDA-MB-468
B
LINC01094 - + +
pcDNA3.1 + - -
TEPP-46 - - +
DMSO + + -
Tetramer
1.00 0.95 1.20
Dimer
1.00 1.87 1.49
GAPDH
MDA-MB-468
C
MDA-MB-231
% Relative of glucose uptake
0μM 5μM 10μM 25μM 50μM 100μM
% Relative of lactate levels
Absorbance(450nm)
0μM
5μM
10μM
25μM **
50μM ***
100μM ***
Time(h)
D
MDA-MB-468
0μM
5μM
10μM
25μM *
50μM **
100μM **
E
MDA-MB-231
0h 6h 12h 24h 48h 72h
0h
6h
12h
24h**
48h***
72h***
F
MDA-MB-468
0h
6h
12h
24h*
48h**
72h**
G
TEPP-46 0μM 5μM 10μM 25μM 50μM 100μM
MDA-MB-231 Migration Invasion
MDA-MB-468 Migration Invasion
Cell Number
Migration Invasion
H
TEPP-46 0h 6h 12h 24h 48h 72h
I
MDA-MB-468
% Relative of glucose uptake
% Relative of lactate levels
pcDNA3.1+DMSO
LINC01094+DMSO
LINC01094+TEPP-46
J
MDA-MB-468
pcDNA3.1+DMSO
LINC01094+DMSO
LINC01094+TEPP-46
ECAR (mpH/min)
Glucose
Oligomycin
2-DG
Time (min)
OCR (pmol/min)
Oligomycin
FCCP
Rotenone & antimycin A
K
MDA-MB-231
MDA-MB-468
pcDNA3.1+DMSO
LINC01094+DMSO
LINC01094+TEPP-46
Relative mRNA expression
LDHA GLUT1 PDK1 PDK4 HK2 PKM2 C-MYC CCND1
L
pcDNA3.1 + - - + - -
LINC01094 - + + - + +
DMSO + + - + + -
TEPP-46 - - + - - +
LDHA 1.00 1.48 1.33 1.00 1.15 0.86
GLUT1 1.00 1.41 1.10 1.00 1.10 0.71
PDK1 1.00 1.16 0.64 1.00 1.10 0.91
PDK4 1.00 1.30 1.05 1.00 1.37 1.08
HK2 1.00 1.39 1.10 1.00 1.26 0.99
PKM2 1.00 1.31 0.97 1.00 1.20 0.91
C-MYC 1.00 1.15 0.93 1.00 1.50 1.05
CCND1 1.00 1.20 0.89 1.00 1.33 1.18
GAPDH
MDA-MB-231 MDA-MB-468

Figure S10. LINC01094 is a potential therapeutic target for BC.

(A) Intracellular examination revealed that elevation of LINC01094 resulted in an increase in PKM2 distribution within the nucleus, yet this effect was notably counteracted by TEPP-46 treatment in MDA-MB-468 cells. **(B)** Western blot assays conducted subsequent to DSS crosslinking experiments illustrated that TEPP-46 efficiently abolished the augmentation of PKM2 dimerization induced by LINC01094 in MDA-MB-468 cells. **(C-H)** The effects of TEPP-46 treatment at different concentrations (0, 5, 10, 25, 50, 100μM) **(C, D and G)** and time intervals (0, 6, 12, 24, 48, 72h) **(E, F and H)** on BC cell energy metabolism and tumor progression were assessed through glucose uptake measurement, lactate production analysis, CCK-8 proliferation assay, and transwell migration/invasion assays. **(I, J)** Glucose uptake and lactate production assays **(I)**, along with ECAR and OCR measurements **(J)**, validated that TEPP-46 could revert the enhanced energy metabolism reprogramming induced by LINC01094 overexpression in MDA-MB-468 cells. **(K, L)** qRT-PCR analyses **(K)** and western blot **(L)** substantiated that TEPP-46 effectively countered the upregulation of downstream genes induced by LINC01094 overexpression in BC cells. Data are presented as mean ± SD; statistical significance is indicated (*p < 0.05; **p < 0.01; ***p < 0.001) by ANOVA.

Supplementary tables

Table S1. Detailed clinicopathological features of patients

Patient ID	Age	Grade	Tumor size (cm)	Number of lymph nodes metastasis	ER	PR	HER2	T staging	pN staging
1	54	I	2	0	Positive	Positive	Negative	T1	N0
2	60	II	2.2	0	Positive	Positive	Positive	T2	N0
3	49	II	1.5	0	Positive	Positive	Negative	T1	N0
4	61	II	4.1	0	Positive	Positive	Positive	T2	N0
5	50	III	2.5	0	Positive	Positive	Negative	T2	N0
6	54	III	2.5	0	Negative	Positive	Positive	T2	N0
7	49	II	3.5	0	Negative	Negative	Positive	T2	N0
8	50	III	2.5	0	Negative	Negative	Positive	T2	N0
9	53	III	2.2	0	Positive	Positive	Negative	T2	N0
10	72	Missing	2	16	Positive	Positive	Negative	T1	N3
11	43	II	2.2	0	Positive	Positive	Positive	T2	N0
12	53	III	2.5	4	Positive	Positive	Positive	T2	N2
13	56	II	3	12	Positive	Positive	Negative	T2	N3
14	60	Missing	2	0	Positive	Positive	Negative	T1	N0
15	57	II	2	0	Positive	Positive	Negative	T1	N0
16	51	III	2.4	0	Positive	Positive	Negative	T2	N0
17	73	III	2.5	0	Positive	Negative	Positive	T2	N0
18	37	III	2	0	Positive	Positive	Positive	T1	N0
19	57	II	2	0	Positive	Positive	Negative	T1	N0
20	48	II	1	0	Positive	Positive	Negative	T1	N0
21	60	II	1.5	1	Positive	Positive	Positive	T1	N1
22	51	II	1.6	1	Positive	Positive	Negative	T1	N1
23	48	II	1.8	0	Positive	Positive	Negative	T1	N0
24	48	II	6	10	Positive	Positive	Negative	T3	N3
25	46	III	2.5	0	Positive	Positive	Negative	T2	N0
26	51	III	2.5	2	Negative	Negative	Negative	T2	N1
27	41	II	5.5	6	Positive	Positive	Negative	T3	N2
28	51	II	1	0	Positive	Negative	Negative	T1	N0
29	36	III	2.5	1	Positive	Positive	Positive	T2	N1
30	47	II	3.8	14	Positive	Positive	Negative	T2	N3
31	50	II	1.8	5	Positive	Positive	Negative	T1	N2
32	43	II	1.5	1	Positive	Positive	Negative	T1	N1
33	64	II	1.8	2	Positive	Positive	Negative	T1	N1
34	67	III	3.2	2	Positive	Positive	Positive	T2	N1
35	50	III	5.5	0	Negative	Negative	Positive	T3	N0
36	47	Missing	2.7	0	Positive	Negative	Positive	Tis	N0

37	55	Missing	3.5	0	Negative	Negative	Positive	Tis	N0
38	37	II	2.4	14	Positive	Positive	Negative	T2	N3
39	34	II	1.5	3	Positive	Positive	Positive	T1	N1
40	75	II	2.8	2	Positive	Positive	Negative	T2	N1
41	51	Missing	2.3	1	Positive	Positive	Positive	Tis	N1
42	53	II	1.5	0	Positive	Negative	Negative	T1	N0
43	70	II	5	4	Positive	Negative	Negative	T2	N2
44	61	III	2.8	1	Positive	Negative	Positive	T2	N1
45	54	II	4	2	Positive	Positive	Negative	T2	N1
46	75	III	6	0	Positive	Positive	Negative	T3	N0
47	50	III	2.2	0	Negative	Negative	Negative	T2	N0
48	54	III	2.1	0	Negative	Negative	Negative	T2	N0
49	44	II	2.5	0	Positive	Positive	Positive	T2	N0
50	46	III	3	1	Negative	Negative	Negative	T2	N1
51	63	III	2.5	0	Positive	Negative	Negative	T2	N0
52	64	Missing	2.5	0	Negative	Negative	Positive	Tis	N0
53	58	Missing	2.5	0	Positive	Positive	Negative	T2	N0
54	64	Missing	2.5	0	Negative	Negative	Positive	Tis	N0
55	34	II	4.6	1	Negative	Positive	Negative	T2	N1
56	45	Missing	5	0	Negative	Negative	Negative	T2	N0
57	42	Missing	3.5	0	Positive	Positive	Negative	T2	N0
58	61	Missing	3.9	6	Missing	Missing	Missing	T2	N2
59	60	II	1.6	1	Positive	Positive	Negative	T1	N1
60	56	III	4	1	Negative	Negative	Negative	T2	N1
61	52	II	1.3	0	Positive	Positive	Positive	T1	N0
62	42	III	1.2	0	Negative	Negative	Negative	T1	N0
63	43	III	3	0	Negative	Negative	Negative	T2	N0
64	37	Missing	4.5	0	Negative	Negative	Positive	Tis	N0
65	37	Missing	4.5	0	Negative	Negative	Positive	Tis	N0
66	43	III	3	0	Negative	Negative	Negative	T2	N0
67	45	III	2.2	1	Negative	Negative	Positive	T2	N1
68	52	Missing	2	0	Positive	Positive	Negative	T1	N0
69	69	Missing	2.5	4	Negative	Negative	Negative	T2	N2
70	57	III	3	15	Positive	Positive	Negative	T2	N3
71	74	II	3.2	4	Positive	Positive	Negative	T2	N2
72	38	III	1.5	0	Positive	Positive	Negative	T1	N0
73	76	II	2	1	Positive	Positive	Negative	T1	N1
74	74	II	3.2	4	Positive	Positive	Negative	T2	N2
75	60	III	3	2	Negative	Negative	Negative	T2	N1
76	48	III	6	7	Positive	Positive	Positive	T3	N2
77	58	Missing	2.2	2	Positive	Positive	Negative	T2	N1
78	62	III	2.2	19	Negative	Negative	Negative	T2	N3

79	38	Missing	3	2	Positive	Positive	Negative	T2	N1
80	40	Missing	3.5	3	Positive	Negative	Negative	T2	N1
81	55	II	2.5	5	Negative	Positive	Negative	T2	N2
82	52	III	3	3	Positive	Positive	Negative	T2	N1
83	57	II	1.1	1	Positive	Positive	Negative	T1	N1
84	49	III	2.2	1	Negative	Negative	Positive	T2	N1
85	61	II	2	1	Negative	Negative	Negative	T1	N1
86	42	Missing	2	2	Positive	Positive	Negative	T1	N1
87	60	II	2.8	5	Negative	Negative	Negative	T2	N2
88	51	I	3	2	Positive	Positive	Negative	T2	N1
89	55	Missing	2.3	1	Missing	Missing	Missing	T2	N1
90	61	II	2	1	Negative	Negative	Negative	T1	N1

Table S2. Gene sequences and antibodies

**Primers for qRT-PCR**	
Genes	Sequences
LINC01094 F	TGCAAAGCAGGTGACTGGAT
LINC01094 R	AACATGGAGTGCCTGCCAAA
PKM2 F	ATTATTTGAGGAACTCCGCCGCCT
PKM2 R	ATTCCGGGTCACAGCAATGATGG
LDHA F	TTGTTGGGGTTGGTGCTGTTG
LDHA R	AAGAGCAAGTTCATCTGCCAAG
GLUT1 F	TGTGTATGCCACCATTGGCT
GLUT1 R	CTAGCGCGATGGTCATGAGT
PDK1 F	GCCACTATGGAACACCATGC
PDK1 R	CCTCATTACCCAGCGTGACA
PDK4 F	GGTGGTGTTCCCCTGAGAAT
PDK4 R	GCAAGCCGTAACCAAAACCA
CCND1 F	GAAGGAGACCATCCCCCTGA
CCND1 R	GAAATCGTGCGGGGTCATTG
CMYC F	CCCCTACCCTCTCAACGACA
CMYC R	CTTCTTGTTCCTCCTCAGAGTCG
**Primers for MeRIP validation**	
Genes	Sequences
LINC01094-m^{6}A-99 F	CACGGATTCGATTTAGGCACG
LINC01094-m^{6}A-99 R	TTCGGATGCCTTTCTGGAGC
LINC01094-m^{6}A-627 F	GAGAATGTCTGCTGGGTTAGC
LINC01094-m^{6}A-627 R	CATGGTGTCTCACTGGGTGAT
LINC01094-m^{6}A-1013+1021 F	GAAACAAGAGCAGCAGCAGAT
LINC01094-m^{6}A-1013+1021 R	TGCCTCATAAGCTGAGTTTCA
LINC01094-m^{6}A-1099+1135 F	TTGGCTAAAGGTCAAGGTGACTTA
LINC01094-m^{6}A-1099+1135 R	GGAGTGCCTGCCAAACAAGA
**Antibodies**	**Source**
LP0	Santa Cruz Biotechnology
PKM2	Proteintech
GAPDH	ZEN BIO
Lamin B1	ZEN BIO
JMJD5	Santa Cruz Biotechnology

HIF-1α	Santa Cruz Biotechnology
GLUT1	Santa Cruz Biotechnology
HK2	Proteintech
LDHA	Santa Cruz Biotechnology
PDK1	Proteintech
PDK4	Santa Cruz Biotechnology
CCND1	Santa Cruz Biotechnology
CMYC	Santa Cruz Biotechnology
